# Impact of Temperature Elevation on Microbial Communities and Antibiotic Degradation in Cold Region Soils of Northeast China

**DOI:** 10.3390/toxics12090667

**Published:** 2024-09-13

**Authors:** Zijun Ni, Xiaorong Zhang, Shuhai Guo, Huaqi Pan, Zongqiang Gong

**Affiliations:** 1 Key Laboratory of Pollution Ecology and Environmental Engineering, Institute of Applied Ecology, Chinese Academy of Sciences, Shenyang 110016, China; nizijun21@mails.ucas.ac.cn (Z.N.); xrzhang@iae.ac.cn (X.Z.); 2 University of Chinese Academy of Sciences, Beijing 100049, China; 3 National-Local Joint Engineering Laboratory of Contaminated Soil Remediation by Bio-Physicochemical Synergistic Process, Institute of Applied Ecology, Chinese Academy of Sciences, Shenyang 110016, China; shuhaiguo@iae.ac.cn; 4 CAS Key Laboratory of Forest Ecology and Silviculture, Institute of Applied Ecology, Chinese Academy of Sciences, Shenyang 110016, China

**Keywords:** greenhouse warming, antibiotic pollution, community assembly, bioremediation, rare taxa

## Abstract

This study systematically investigated the effects of temperature changes on the degradation of antibiotics in soil, as well as the alterations in microbial community structure and aggregation, through a field warming experiment in a greenhouse. Compared to non-warming soil, the warming treatment significantly accelerated the degradation rate of tetracyclines during soil freezing and mitigated the impact of environmental fluctuations on soil microbial communities. The greenhouse environment promoted the growth and reproduction of a wide range of microbial taxa, but the abundance of Myxococcota was positively correlated with antibiotic concentrations in both treatments, suggesting a potential specific association with antibiotic degradation processes. Long-term warming in the greenhouse led to a shift in the assembly process of soil microbial communities, with a decrease in dispersal limitation and an increase in the drift process. Furthermore, co-occurrence network analysis revealed a more loosely structured microbial community in the greenhouse soil, along with the emergence of new characteristic taxa. Notably, more than 60% of the key taxa that connected the co-occurrence networks in both groups belonged to rare taxa, indicating that rare taxa play a crucial role in maintaining community structure and function.

## 1. Introduction

The widespread and often excessive use of antibiotics in agriculture has led to increasing levels of antibiotic residues in rural environments, posing significant threats to ecosystems and human health. Agricultural activities, such as livestock farming and aquaculture, along with human medical waste disposal, contribute to the diverse sources and complex pathways of antibiotic pollution in agricultural soils [1,2]. In northern China, the average contamination levels of tetracycline and enrofloxacin in soils around small-scale farms were 51.32–299.7 μg kg^−1^ and 63.55–197.4 μg kg^−1^, respectively, primarily concentrated in the 0–10 cm soil layer [3]. The degradation pathways and half-lives of these antibiotics in the environment vary depending on their chemical structures and environmental conditions [4]. Long-term, complex pollution can also lead to the reverse transformation of metabolites, adversely affecting the activity, composition, and function of soil microbial communities [5,6].

Bioremediation using indigenous microorganisms is a common method for remediating agricultural soil contamination, offering advantages such as environmental friendliness, economic feasibility, long-term effectiveness, ease of operation, and high social acceptance [7,8,9]. However, northern regions experience a three-month-long frozen soil period each year, causing significant changes in the abundance and structure of soil microbial communities and thus weakening the effectiveness of bioremediation. Therefore, increasing soil temperature during the frozen period could be a potential mitigation strategy. For instance, studies have shown that covering the surface of hydrocarbon-contaminated soil in Antarctica with a specialized black film can effectively increase the average soil temperature and promote contaminant removal [10]. Given the climatic characteristics of northern regions, greenhouses have been widely used to improve crop growth rates. As of 2016, the area covered by greenhouses in northern China had reached 13.15 million hectares [11]. Greenhouse warming can extend the period of microbial activity and promote the sustainable development of agriculture. However, systematic studies on the promotion of bioremediation of antibiotic-contaminated agricultural soils by winter warming and the succession and assembly of soil microbial communities in greenhouses are scarce. Additionally, previous studies on the relationship between soil microbial community structure and function and pollutants have largely focused on the response of high-abundance microbial taxa [12,13]. This may be because high-abundance taxa have broader ecological niches in the community, endowing them with stronger environmental adaptability [14]. However, recent studies have increasingly recognized the importance of the high diversity and functional redundancy of rare microbial taxa in maintaining community functional stability [15,16,17]. Therefore, a combined analysis of abundant and rare taxa will help us better understand the impacts of pollutant stress on soil microbial community structure and function, as well as the interactions between different abundance classes of microbes during the remediation process.

This study, using low-cost greenhouses commonly found in northern rural areas as an experimental platform, aims to systematically investigate the effects of greenhouse warming on the bioremediation of complex antibiotic-contaminated agricultural soils in northern regions. Through year-long field monitoring, we compared the antibiotic degradation rates of agricultural soils under greenhouse warming and non-warming treatments, and combined the dynamic changes in soil microbial community structure to reveal: (1) the promotion of bioremediation of antibiotic-contaminated agricultural soils in northern regions by greenhouse warming; (2) impact of greenhouse warming on the stability of soil microbial communities; and (3) role of rare microbial taxa in antibiotic degradation processes. By comprehensively analyzing both abundant and rare microbial taxa, this study will help elucidate their roles in the bioremediation of antibiotic-contaminated agricultural soils and provide theoretical and practical support for bioremediation of soil pollutants in northern regions.

## 2. Materials and Methods

### 2.1. Site Description and Experimental Design

**Figure 1:**
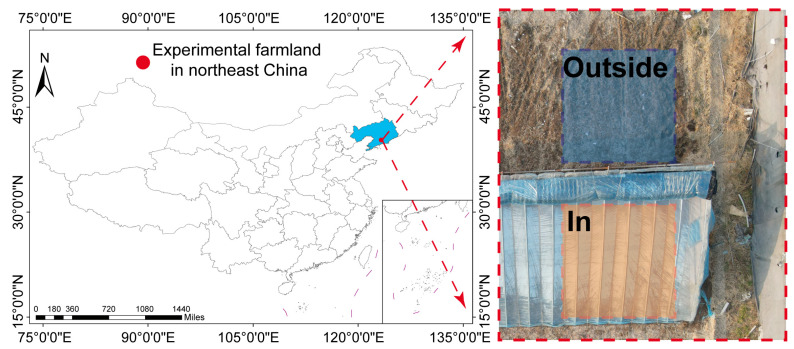
Location and experimental layout of the antibiotics contaminate farmland.

The antibiotic-contaminated farmland was located in Chaoyang Town, Xiuyan Manchu Autonomous County, Anshan City, Liaoning Province, China (123°33′36″ E, 40°48′12″ N) (Figure 1). The farmland had been used for chicken manure storage for approximately four years, with dimensions of approximately 25 m × 35 m. The soil was contaminated with tetracyclines at concentrations ranging from 841.2 to 2342.7 μg kg^−1^ and with fluoroquinolones at concentrations ranging from 1035.7 to 2415.5 μg kg^−1^. The sandy loam agricultural soil had a particle size distribution of 8.9% (2–0.2 mm), 48.8% (0.2–0.02 mm), 29.4% (0.02–0.002 mm), and 12.9% (<0.002 mm); a pH of 6.34; an organic matter content of 21.6 g kg^−1^; a total nitrogen content of 1.37 g kg^−1^; an electrical conductivity of 0.54 mS cm^−1^; and a cation exchange capacity of 8.63 mmol kg^−1^. The lowest monthly average temperature occurs consistently in January (−15 °C), with the highest typically occurring in July or August (28 °C).

The topsoil (0.5 m depth) within a 6 m × 5 m area was homogenized and subsequently divided into two treatments (In, Outside), each measuring 2.5 m × 2.5 m, with a 1 m interval between treatments to ensure data independence (Figure 1). Each treatment consisted of three replicates. Five-point sampling was conducted in each treatment area, and the samples were mixed to obtain T0 samples for both treatments. A greenhouse (5 m × 30 m × 2.5 m) was constructed above the In treatment, with 35 cm-thick grass bricks stacked on the north side for wind protection and a thick fabric covering the plastic film for nighttime insulation. The fabric was raised to the top of the greenhouse from 10:00 a.m. to 3:00 p.m. to increase the temperature, while the greenhouse door was opened to ventilate the In treatment area (from November to April). During other months, the door was kept open to maintain ventilation, and the thick fabric was not raised from 10:00 a.m. to 3:00 p.m. to reduce the greenhouse warming effect and prevent excessive temperature differences between the two treatments in summer. Soil temperature monitoring instruments were placed approximately 5 cm below the soil surface, and soil daily average temperature was calculated based on data transmitted every hour. Soil samples were collected monthly for a period of twelve months, spanning from October of the first year to October of the second year. Within each month, samples were obtained on a single day during the final week of the month from both treatment areas. A five-point sampling method was employed to collect samples (3–5 cm below the topsoil, approximately 1 kg). These monthly samples were designated as T1 through T12, with T1 representing November of the first year and T12 representing October of the second year. The initial soil sample, collected prior to the commencement of the experiment, was designated as T0. Samples were transported to the laboratory at 4 °C and divided into two parts, both stored at −80 °C. One part was used for antibiotic concentration determination, and the other part was used for DNA extraction. For DNA extraction, only samples from T0, T4 (February of the second year), T8 (June of the second year), and T12 were utilized. All collected samples were filtered through a 100-mesh nylon sieve to remove plant roots and gravel before analysis.

### 2.2. Extraction and Quantification of Antibiotics

A measure of 2 (2 ± 0.001) g of homogenized dry soil was accurately weighed into a 50 mL centrifuge tube, and 5 mL acetonitrile and 5 mL Na2EDTA-McIlvaine solution were added. The mixture was vortexed for 2 min, shaken for 30 min, and ultrasonically extracted for 10 min. After centrifugation at 8000 rpm for 10 min, the extraction was repeated twice. The three extracts were combined, mixed, and diluted with ultrapure water to 400 mL. The pH of the extract was adjusted to 2.9 ± 0.1 with formic acid. The extract was purified and enriched using a pre-activated SAX-HLB tandem solid-phase extraction column at a flow rate of 3–4 mL/min. After passing through the column, the SAX-HLB tandem solid-phase extraction column was washed with 6 mL of ultrapure water, and the SAX column was removed. The HLB extraction column was dried under vacuum for 30 min to remove residual water. Finally, the column was eluted with 6 mL of methanol, and the eluate was collected and concentrated to near dryness using a gentle nitrogen stream. The residue was redissolved in 1 mL of methanol, vortexed for 30 s, mixed well, filtered through a 0.22 μm filter into a 2 mL brown liquid chromatography vial, and then stored at −20 °C in the dark [18,19] for determination using ultra-high performance liquid chromatography–triple quadrupole mass spectrometry (Triple Quad 5500+ QTRAP Ready, SCIEX, Framingham, MA, USA). The preparation of Na2EDTA-McIlvaine solution, the activation of SAX-HLB tandem solid-phase extraction columns, and the quantification and recovery of antibiotics are provided in the Supplementary Materials (Texts S1 and S2, Table S1).

### 2.3. Biological Analysis

A total of 24 soil samples from the In and Outside treatments at T0, T4, T8, and T12 were subjected to high-throughput sequencing. High-throughput sequencing was performed by Personalbio Ltd. (Shanghai, China). The hypervariable V3-V4 region of the 16S rRNA gene was amplified [20]. After purification, the PCR products were subjected to paired-end sequencing on an Illumina HiSeq2500 PE250 platform (San Diego, CA, USA). The DADA2 method [21] was used for quality control, including primer removal, quality filtering, and chimera removal, to obtain ASV feature sequences and perform taxonomic annotation. An ASV abundance and species annotation table was created, and based on this table, microbial community structure changes were analyzed. Abundant and rare taxa were divided according to the relative abundance of ASVs. ASVs with a relative abundance greater than 0.1% in any experimental sample were considered abundant taxa, while ASVs with a relative abundance not exceeding 0.1% in all experimental samples were considered rare taxa [16,17].

### 2.4. Computational Analyses

Unless otherwise stated, all statistical analyses were performed using R 4.2.2. The Wilcoxon rank-sum test and t-test were conducted using the ‘ggpubr’ package to compare differences between two groups. Shannon and Chao 1 indices, as well as non-metric multidimensional scaling (NMDS) based on Bray–Curtis distances, were calculated using the ‘vegan’ package [22]. The relative importance of different ecological processes in bacterial community assembly was calculated using null model analysis in the ‘iCAMP’ software package [23]. Specifically, the entire community was divided into “bins” of 12–48 phylogenetically related ASVs, and the relative importance of five community assembly processes for each bin was calculated: heterogeneous selection, homogeneous selection, dispersal limitation, homogeneous dispersal, and drift. The relative contribution of each ecological process to community assembly in each treatment was calculated based on the ecological processes and relative frequencies of each bin. The results were visualized using the ‘itol.toolkit’ package and “iTOL” web-based tool [24,25]. Microbial co-occurrence networks were constructed based on Spearman correlations between ASVs using the ‘WGCNA’ package [26]. The ‘ggClusterNet’ package [27] was used to calculate “within modular degree” (Pi) and “between modular degree” (Zi) to identify key taxa in the co-occurrence network. The association pairs were visualized as a co-occurrence network using the Fruchterman–Reingold layout in Gephi 0.9.7. Random forest models were generated and analyzed using the ‘randomForest’ package.

### 2.5. Statistical Analysis

Experimental data were processed using Excel 2010 (Microsoft, Redmond, WA, USA), and the results were presented as mean ± standard deviation. Kruskal–Wallis tests were conducted using SPSS 19.0 (SPSS Inc., Chicago, IL, USA) to analyze differences among multiple groups. Some figures were visualized using ‘ggplot2’ in R.

## 3. Results

### 3.1. Changes in Soil Antibiotic Degradation Rates under Different Temperature Conditions

**Figure 2:**
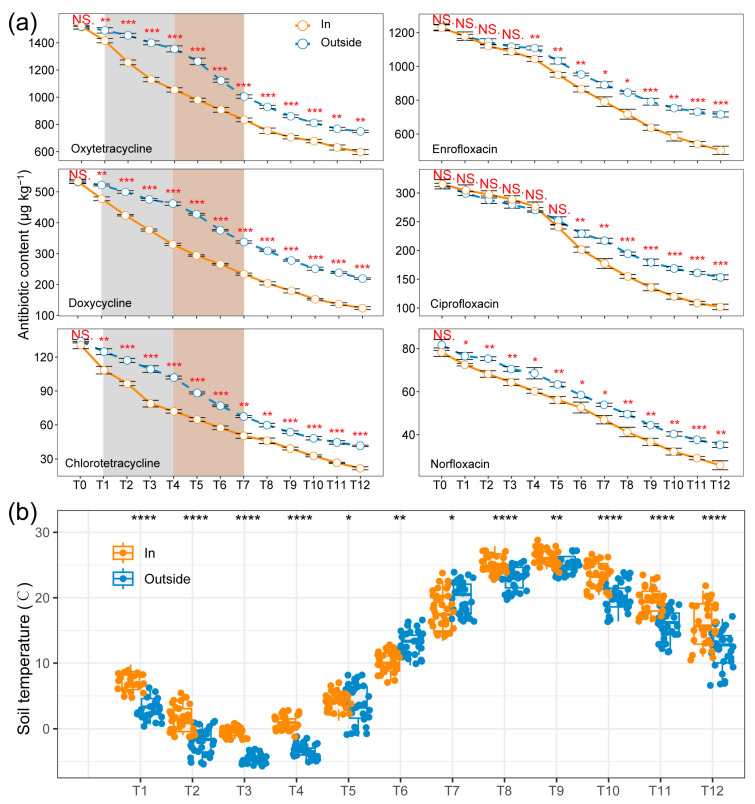
Changes in antibiotic concentration (**a**) and soil temperature (**b**) in In and Outside treatments across twelve months. * *p* < 0.05, ** *p* < 0.01, *** *p* < 0.001, **** *p* < 0.0001, NS, not significant.

Figure 2a and Figure S1 illustrate the changes in soil residues and degradation rates of six antibiotics over a 12-month period under both warming and non-warming conditions. Overall, the residues showed a decreasing trend, with the total degradation rate of antibiotics being significantly higher in the warming treatment compared to the non-warming treatment (Table 1, Figure S1). Between T1 and T5, the degradation rate gap between the two treatments progressively increased for tetracyclines (Oxytetracycline: 5.2–20%; Doxycycline: 8.6–25.3%; Chlortetracycline: 10.1–21.3%), before decreasing from T5 to T10 (Oxytetracycline: 20–8.9%; Doxycycline: 25.3–18.6%; Chlortetracycline: 21.3–9.5%; Table S2). In contrast, for fluoroquinolones (excluding low-content Norfloxacin), no significant difference in the soil residues of Enrofloxacin and Ciprofloxacin was observed between the two treatments during T0-T3 and T0-T5, respectively (*p* > 0.05; Figure 2a), and no significant difference in their degradation rates was found at T1 or between T1 and T5 (*p* > 0.05; Figure S1). Figure 2b presents the daily average soil temperature changes over 12 months in both treatments. Except for months 5 to 7, the soil temperature in the warming treatment (In) was significantly higher than that in the non-warming treatment (Outside). In the third month (January), the soil temperature was the lowest throughout the year, with the soil in the In treatment undergoing freeze–thaw cycles and the soil in the Outside treatment being completely frozen, with a minimum temperature of −5.9 °C. In the ninth month (July), the soil temperature reached the highest point of the year, with the maximum temperature in the In treatment being 29.1 °C.

### 3.2. Effects of Temperature Differences on Bacterial Community Succession

**Figure 3:**
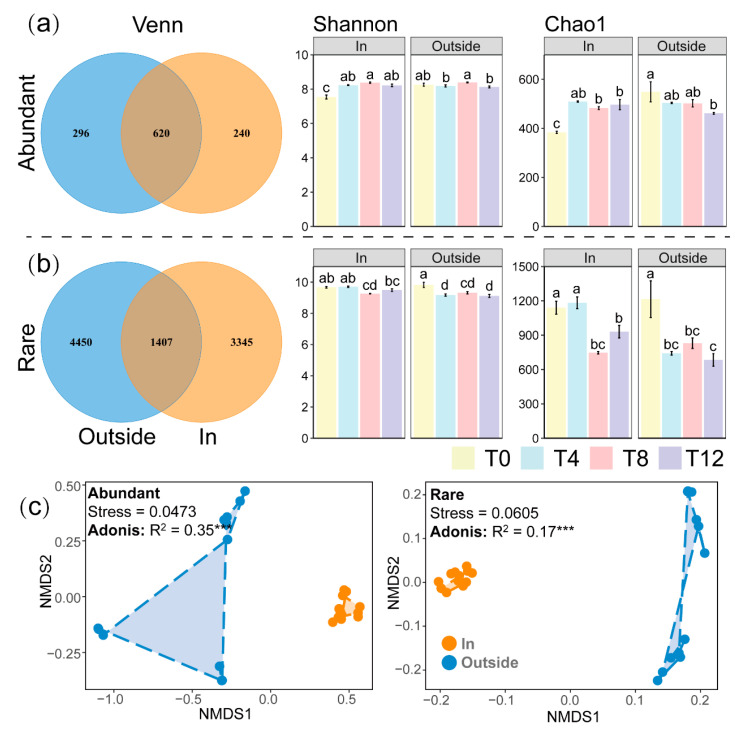
Diversity analysis of In and Outside treatments. Venn diagrams of the abundant (**a**) and rare (**b**) communities in both treatments, along with Shannon and Chao1 indices at four time points. (**c**) NMDS analysis of community structure based on Bray–Curtis distances for both abundant and rare categories. *** *p* < 0.001, a, b, c, and d reflect the 5% significance level.

A total of 10,358 bacterial ASVs were identified in all samples, including 1156 abundant species and 9202 rare species. Venn diagrams show the differences in the composition of abundant and rare taxa between the two treatments (Figure 3a,b). Among abundant taxa, the number of shared ASVs (620) between the two treatments was more than twice the number of independent ASVs (Outside: 296, In: 240); conversely, for rare taxa, the number of shared ASVs (1407) was much lower than the number of independent ASVs (Outside: 4450, In: 3345). This indicates that long-term environmental differences had a smaller impact on the species diversity of abundant taxa, which helped to stabilize the basic soil functions. In contrast, the competition and predation processes of rare microbial communities were significantly affected.

To further explore the effects of temperature differences on the differences in α-diversity of microbial communities, we conducted α-diversity analyses on both abundant and rare taxa (Figure 3a,b). In the warming system, the α-diversity indices (Shannon and Chao1) of abundant taxa were lowest at T0 (*p* < 0.05), and the Shannon and Chao1 indices of rare taxa were lowest in summer (T8) (*p* < 0.05). This suggests that in the semi-closed warming system, as pollutant stress decreased, the microbial community gradually evolved from functionally unstable rare taxa to higher abundance taxa, increasing community stability. In the non-warming system, both α-diversity indices were highest at T0 (*p* < 0.05) and lowest at T12 (*p* < 0.05), with the α-diversity indices of rare taxa decreasing significantly at T4, T8, and T12 (*p* < 0.05), indicating that rare bacterial communities in open environments were more sensitive to environmental and temperature changes, rather than changes in pollutant concentrations.

NMDS analysis of Asonis (Figure 3c) showed that temperature differences resulted in significant changes in bacterial community structure for both abundant and rare taxa, but the In treatment was more concentrated. Compared with the Outside treatment, the In treatment, due to its location inside the greenhouse, had a more stable semi-closed environment, with reduced resource limitations and external inputs, resulting in smaller-scale succession of microbial communities. In contrast, natural changes, external inputs, and resource fluctuations in the open environment led to greater succession amplitudes in microbial communities.

### 3.3. Differential Responses of Antibiotic-Responsive Bacterial Populations to Temperature

**Figure 4:**
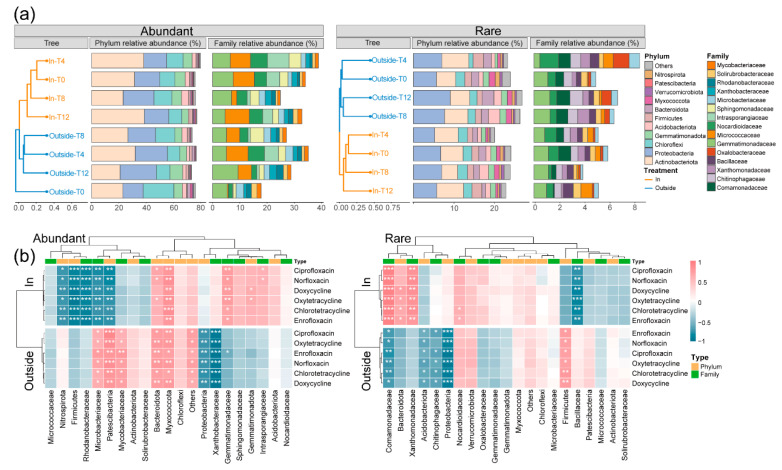
Analysis of the correlation between dominant phyla and families with antibiotics. Abundance changes in the top ten phyla and families in the abundant and rare communities of the In and Outside treatments at four time points (**a**), and the Spearman correlation with six antibiotics (**b**). * *p* < 0.05, ** *p* < 0.01, *** *p* < 0.001.

To further investigate the differential responses of bacterial populations to antibiotics under different temperature conditions, phylogenetic analyses were conducted on ASV species identified in both treatments, separately for abundant and rare categories. The top ten dominant phyla and families in each category were screened, and their relative abundance changes over four time points were presented. In conjunction with the corresponding residual concentrations of six antibiotics, the different responses of bacterial communities to antibiotic contamination under the influence of temperature were analyzed (Figure 4a,b, Table S3).

In the abundant category, Actinobacteriota (Outside: 25.46%, In: 32.78%), Proteobacteria (Outside: 21.38%, In: 18.94%), Chloroflexi (Outside: 13.92%, In: 11.46%), and Gemmatimonadota (Outside: 6.5%, In: 6.53%) had relatively high average abundances. Actinobacteriota and Proteobacteria also had high abundances in the rare category, indicating that these two phyla played important roles in community evolution and accounted for a total abundance of 61.55%. However, the relative abundances of these two phyla fluctuated at different time points. The abundance of Actinobacteriota at T4 (temperature < 0 °C) was much higher than that at T8 (temperature > 20 °C) in both treatments and both bacterial groups, indicating that Actinobacteriota had high activity in low-temperature soil. In contrast, the abundance of Proteobacteria showed an upward trend in the Outside treatment and a fluctuating trend in the In treatment. Additionally, Acidobacteriota, Gemmatimonadota, and Myxococcota in the rare category showed different abundance trends compared to Actinobacteriota, with high abundance only at T8 under ambient temperature. Interestingly, compared to the abundant category, Firmicutes, Bacteroidota, Myxococcota, Verrucomicrobiota, and Patescibacteria had significantly higher abundances in the rare category. The abundance trends of Firmicutes and Bacteroidota were opposite in the warming and non-warming systems. The relative abundance of Bacteroidota increased in the Outside treatment, while the relative abundance of Firmicutes decreased. In the In treatment, the abundance of Bacteroidota decreased, while the abundance of Firmicutes increased.

Analyzing the dominant families in both treatments, the total relative abundance at T4 was higher than that at T8 in both abundant and rare categories, indicating a higher microbial succession rate in frozen soil compared to summer ambient temperature soil. In the abundant category, Intrasporangiaceae (Actinobacteriota) and Micrococcaceae (Actinobacteriota) had high abundance at T4 in both groups; Microbacteriaceae (Actinobacteriota) and Mycobacteriaceae (Actinobacteriota) showed a decreasing trend in abundance in Outside soil, while Xanthobacteraceae (Proteobacteria) showed an increasing trend, indicating that these three dominant families were insensitive to temperature changes. Unlike the dominant families with regular changes in the abundant category, which belonged to the Outside treatment, the dominant families with regular changes in the rare category belonged to the In treatment, such as the continuously decreasing Comamonadaceae (Proteobacteria), Oxalobacteraceae (Proteobacteria) and significantly increasing Bacillaceae (Firmicutes). The emergence of this difference may be attributed to the narrow ecological niche of low-abundance bacteria, making them more vulnerable to the drastic environmental fluctuations in the Outside treatment, which introduces uncertainties in their succession process. In contrast, the semi-closed environment under warming conditions provided greater stability, facilitating the steady development of rare bacterial communities.

In the correlation analysis between the abundant community and antibiotics (Figure 4b), the number of dominant phyla (Nitrospirota, Firmicutes, and Patescibacteria) and dominant families (Rhodanobacteraceae and Microbacteriaceae) in the In treatment that were negatively correlated with the concentrations of six antibiotics was higher than that in the Outside treatment (Proteobacteria and Xanthobacteraceae). In contrast, in the Outside treatment, the categories and number of phyla and families (Patescibacteria, Bacteroidota, Myxococcota, Microbacteriaceae, and Mycobacteriaceae) that were positively correlated with the concentrations of six antibiotics were higher than those in the In treatment (Myxococcota and Gemmatimonadaceae). In the rare community, the categories correlated with antibiotic degradation were significantly reduced, with only Bacillaceae (In), Comamonadaceae (Outside), Acidobacteriota (Outside), and Proteobacteria (Outside) being negatively correlated with antibiotic concentrations and Comamonadaceae (In), Xanthomonadaceae (In), and Firmicutes (Outside) being positively correlated.

### 3.4. Microbial Community Assembly

**Figure 5:**
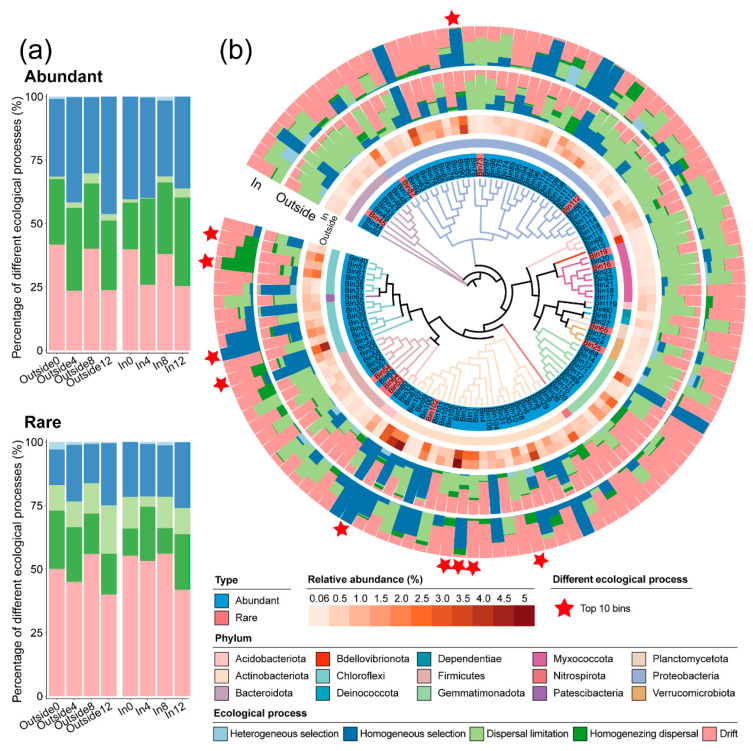
Contribution of community assembly to community aggregation in different treatments. The relative importance of different ecological processes for the rare and abundant communities at four time points (**a**), and the 121 bins (**b**) in In and Outside treatments.

Based on iCAMP results, the relative importance of stochastic and deterministic processes in the abundant and rare taxon communities of the Outside and In treatments showed no significant differences (Figure 5a, Table S4). Both treatments were dominated by homogeneous selection for deterministic processes and by drift for stochastic processes. However, the relative importance of stochastic processes in rare taxon communities (Outside: 79.6%; In: 77.3%) was significantly higher than that in abundant communities (Outside: 62.5%; In: 63.0%), and the relative importance of dispersal limitation, a stochastic process, also increased. Analysis of community assembly in both categories at four time points revealed that, except for T0, the contribution of homogeneous selection was lowest at T8, while the contribution of drift was highest at T8.

The observed 10,358 ASVs were divided into 121 phylogenetic bins. The phylum, relative abundance, and relative importance of each ecological process in the two treatments for ASVs that dominated the abundant community in each phylogenetic bin are shown in Figure 5b. The number and total abundance of bins dominated by each ecological process in the two groups are shown in Table 2. The In treatment significantly increased the proportion of drift in the stochastic process (Figure S2; *p* < 0.05) and significantly decreased the role of dispersal limitation (Figure S2; *p* < 0.05). This indicates that some ASVs adjusted the main ecological processes in bacterial community assembly in response to long-term temperature differences. This transition involved 58 bins (Table S5). Analysis of the top 10 high-abundance bins revealed that warming caused the dominant bacteria in Bin89 (*Devosia*), Bin107 (*Arthrobacter*), Bin1 (*Mycobacterium*), and Bin34 (*KD4-96*) to shift from the stochastic process of drift to the deterministic process of homogeneous selection in the Outside treatment (Table S5). In contrast, Bin3 (*Nocardioides*), Bin2 (*g__unclassified_Actinobacteria*), Bin51 (*AKYG1722*), and Bin30 (*Iamia*) shifted from the stochastic process of dispersal limitation to drift, while Bin33 (*Gitt-GS-136*) primarily shifted from homogeneous selection to drift. This also corroborates the significant advantage of the drift ecological process in the In treatment.

### 3.5. Co-Occurrence Patterns and Key Taxa Analysis of Microbial Communities

**Figure 6:**
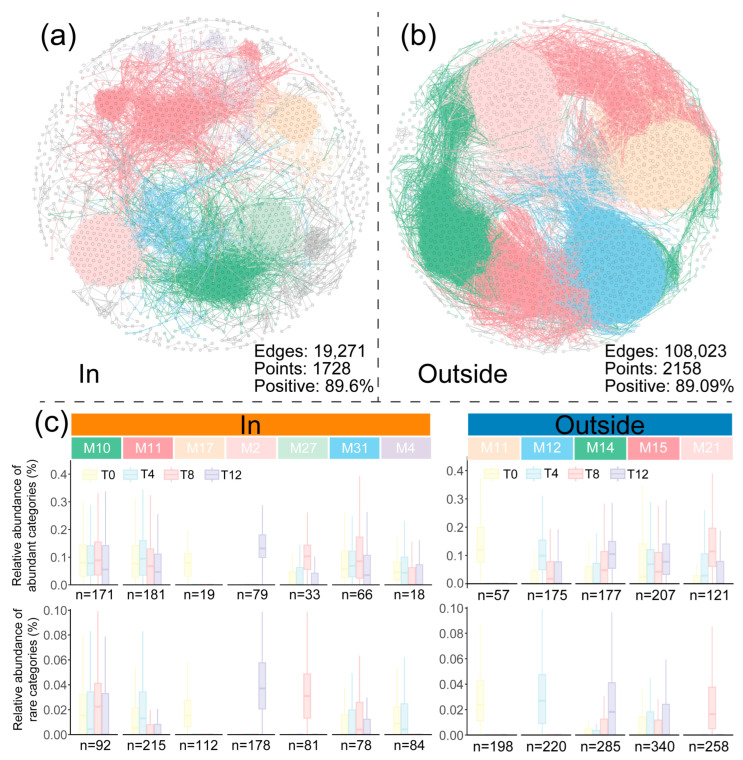
Co-occurrence networks and module abundance analysis. (**a**) Microbial community network co-occurrence analysis for In (**a**) and Outside (**b**) treatments. Two nodes with Spearman’s r > 0.8 and *p* < 0.05 were connected as edges, displaying edges with positive correlations. Nodes connected by edges of the same color belong to the same module. (**c**) Relative abundance changes and ASV numbers of different abundance categories in modules at four time points.

Co-occurrence networks were constructed to explore the succession dynamics of microbial communities in polluted soil under long-term warming and visualize the assembly of abundant and rare taxa (Figure 6a,b). The number of edges and nodes in the In treatment was significantly lower than that in the Outside treatment, which is related to the semi-closed environment of the In treatment. Notably, the number of edges in the Outside treatment was approximately five times that in the In treatment, reflecting the complex interactions of soil microorganisms during long-term freeze–thaw cycles. Figure 6c shows the relative abundance of abundant and rare ASVs contained in highly aggregated modules (number of nodes > 5%) at four time points in both treatments. The ASVs contained in modules M10, M27, and M31 in the In treatment preferred high-temperature environments, while M11 and M4, on the contrary, had stronger survival abilities in soil environments below 0 degrees Celsius. The microorganisms aggregated in M17 and M2 had unique characteristics, which may be attributed to their inability to tolerate sudden environmental changes (M17) or their adaptive evolution due to the significant reduction in pollutants or environmental stability (M2). Similarly, in the Outside treatment, thermophilic microorganisms were mainly aggregated in module M21, while M12 mainly aggregated bacterial communities that could survive in frozen environments. The aggregation of M11 (Outside) was similar to that of M17 (In), but no module similar to M2 (In) was found. In addition, in the Outside treatment, the psychrophilic modules displayed a higher total number of ASVs (peak at T4: 395) relative to the thermophilic modules (peak at T8: 379). However, the In treatment exhibited the opposite pattern, with a higher total number of ASVs in the thermophilic modules (T8: 521) compared to the psychrophilic modules (T4: 498).

**Figure 7:**
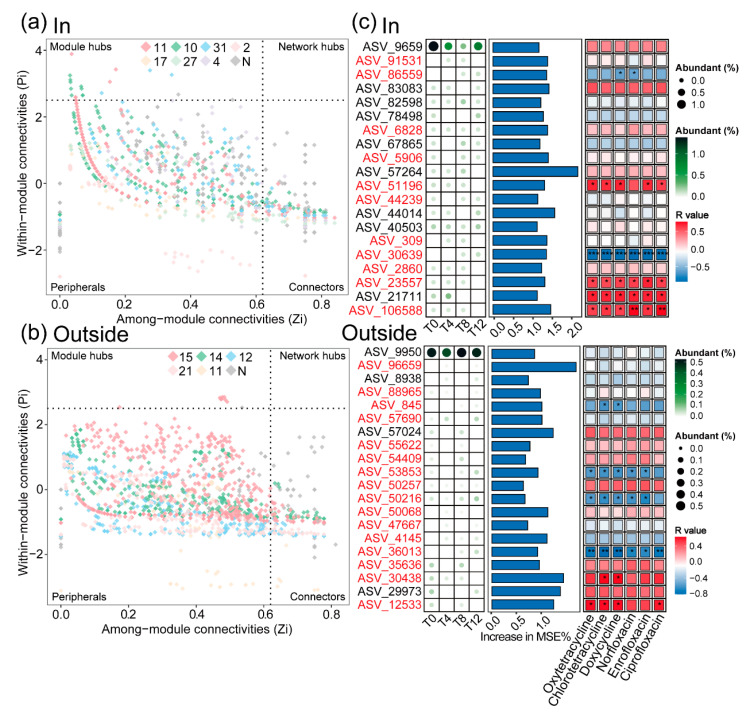
Screening of key taxa in bacterial communities. Zi-Pi plots for In (**a**) and Outside (**b**) treatments. (**c**) The top 20 taxa ranked by feature importance in the random forest model in the two treatments and the correlation with antibiotics. * *p* < 0.05, ** *p* < 0.01, *** *p* < 0.001.

To identify key bacterial taxa playing a crucial role in microbial community assembly, we identified ASVs with connector and module hub roles in both treatments based on Zi-Pi plots (Figure 7a,b, Tables S6–S8). A total of 361 ASVs were identified in the In treatment and 210 in the Outside treatment. We selected the top 20 ASVs with the highest explanatory power for community differences using a random forest model and analyzed their corresponding modules, relative abundances, and correlations with pollutants to compare the differences between key species in the two treatments under long-term warming (Figure 7c, Tables S9 and S10). In the In treatment, 11 ASVs belonged to the rare category, while 16 belonged to the rare category in the Outside treatment. ASVs correlated with antibiotic concentrations mainly belonged to the rare category.

## 4. Discussion

### 4.1. Greenhouse Warming Promotes the Stable Development of Soil Microbial Communities and Accelerates Antibiotic Degradation

A comparison of the temperature differences and antibiotic degradation rates between the warming and non-warming systems over 12 months revealed that greenhouse warming effectively reduced the soil freezing time in northern winters (Figure 2a,b, Figure S1, Table S2). The resulting thermal protection prevented some microbial populations from perishing due to prolonged soil freezing, which favored the natural attenuation of antibiotics [10]. The rapid decline in tetracyclines in the In treatment during the T1–T4 period is a clear example of this. As the soil temperature in the Outside treatment returned to above zero at T5 (March), the gap in tetracycline degradation rates between the Outside and In treatments began to decrease. This suggests that the microorganisms involved in the degradation of tetracyclines are more sensitive to temperature. However, the degradation of Enrofloxacin and Ciprofloxacin did not seem to be favored by psychrophilic bacteria, and there was no significant difference in their residual concentrations when both soils were in a freeze–thaw or frozen state. Studies have shown that this difference is related to their chemical structure. Tetracyclines have a relatively unstable molecular structure and are easily degraded by environmental factors such as light, pH, and temperature. The multiple hydroxyl and carbonyl groups in the molecule readily react with metal ions or organic matter in the soil, accelerating their degradation [28]. In contrast, fluoroquinolones have a more stable molecular structure and are more chemically inert. The fluorine atoms and cyclic structure in their core structure give them a higher resistance to environmental changes and make them less susceptible to degradation [29]. As the soil in the In treatment thawed first, the degradation rates of Enrofloxacin and Ciprofloxacin increased significantly and began to differ from the Outside treatment. Therefore, the early optimization of the soil environment by warming contributes to enhancing the degradation potential of microbial communities and achieving higher antibiotic removal rates.

To investigate the role of abundant and rare taxa in the soil microbial community during antibiotic biodegradation and their response to environmental differences caused by warming, Venn diagram analysis of the microbial community revealed (Figure 3a,b) that the number of bacterial community compositions in the abundant taxa was much lower than that in the rare taxa. However, due to their high abundance and large scale, they have strong resistance to different environmental differences [30]. Low-abundance rare taxa have lower viability and are easily affected by environmental disturbances, leading to changes in composition and community structure [15]. The number of independent ASVs in the In treatment was lower than that in the Outside treatment, which may be due to the fact that the Outside treatment was in an open environment, where temperature changes and climatic influences were more drastic, expanding the range of microbial community succession. The changes in Shannon and Chao1 indices of both abundant and rare categories at four time points in the In treatment, as well as NMDS analysis, also indicate that greenhouse warming can reduce the impact of environmental changes on microbial communities (Figure 3a–c). Stable microbial communities play an important role in maintaining soil nutrient cycling, soil structure, and effective pollutant degradation functions [31,32].

The changes in the abundance of dominant phyla and families in different categories of the two treatments further demonstrated the impact of warming (Figure 4a, Table S3). The relative abundance of the highly abundant Actinobacteriota was more influenced by external air temperature. Studies have shown that microorganisms in Actinobacteriota are cold-tolerant, able to survive and remain active in low-temperature environments, and the ecological competition in winter soil is weaker, allowing them to rapidly proliferate in frozen soil [33,34]. In contrast, the relative abundance of the other major phylum, Proteobacteria, showed an upward trend in the non-warming environment but fluctuated in the warming environment. This may be due to the reduced resource input into the soil caused by the semi-closed environment of the greenhouse, leading to resource limitations and more local competition, resulting in frequent fluctuations in community structure and abundance. Additionally, the relative abundance of some lower-abundance phyla was higher in the rare category than in the abundant category. Interestingly, Firmicutes and Bacteroidota, among them, were found to have a successional change trend during the degradation of straw in paddy soil, with Bacteroidota having a high abundance in the early stage and participating in the initial degradation, followed by the rapid reproduction and succession of Firmicutes in the later stage [35]. Our study also found that the relative abundance changes in the two phyla were opposite in different treatments. Combined with the relative abundance changes in several dominant families, it can be confirmed that the abundance change characteristics of dominant phyla are also reflected in their corresponding dominant families.

Further analysis of changes in antibiotic concentrations, along with the abundance and classification of dominant phyla and families correlated with antibiotics in both treatments, revealed that warming significantly enhanced negative correlations between certain microbial taxa and antibiotic degradation (Figure 4b). Specifically, the abundance of some taxa, including low-abundance phyla and dominant families (e.g., Nitrospirota, Firmicutes, Patescibacteria, Rhodanobacteraceae, Microbacteriaceae), increased as pollutant concentrations decreased. In contrast, under non-warming conditions, the relative abundance of low-abundance phyla declined as antibiotic concentrations diminished. Interestingly, the abundance of Myxococcota decreased in both treatments, potentially due to its involvement in antibiotic degradation. Previous studies have shown that pollutants can influence the abundance of functional bacteria [36]. For example, Wang et al. reported a positive correlation between Mycobacteria abundance and pyrene concentration [31]. Consequently, rare bacteria may develop mechanisms distinct from those of abundant bacteria in response to environmental changes. Thus, abundance differences among microorganisms should not be overlooked when interpreting microbial community succession and function.

### 4.2. Warming Enhanced the Proportion of Stochasticity in Community Assembly and the Independence of Bacterial Communities

Although the abundance analysis of specific microbial taxa helps to understand the response of microbial communities related to warming or antibiotic degradation during community succession, how greenhouse warming affects bacterial community assembly processes remains unclear. Phylogenetic-based null model analysis (iCAMP) was used to study the assembly processes of bacterial communities, quantifying the changes in the relative importance of factors influencing microbial community assembly [23]. The study found that warming did not cause differences in the assembly of abundant and rare-taxa bacterial communities between the two treatments, but the relative importance of stochastic assembly in rare-taxa bacterial communities was higher in both treatments (Figure 5a, Table S4), indicating that changes in rare taxa could affect the stochastic processes of bacterial communities. This result is consistent with the findings of Wang et al. [16].

Analysis of the assembly process of the entire soil community revealed that warming led to a shift in bacterial communities from dispersal limitation to drift (both stochastic processes), but there was no significant change in the deterministic process (Figure S2). Studies have shown that stochastic processes, especially dispersal limitation, are the main factors controlling bacterial community assembly [37]. The decrease in the relative importance of dispersal limitation in the In treatment indicates that the greenhouse environment reduced the dispersal limitations between communities, while the long-term freezing of soil in the Outside treatment to some extent affected bacterial dispersal, leading to a higher proportion of dispersal limitation in the In treatment. The drift process of bacterial communities is usually a random event, and researchers have various analyses regarding the reasons for the increase in the proportion of drift. For example, drift is more prevalent in relatively low-pollution soils, or drastic environmental changes caused by the melting of permafrost can increase the proportion of drift [12,38]. However, Ning et al. believe that ecological drift is a core concept in ecology, and its occurrence is greatly influenced by factors such as selection, dispersal, and diversity [23]. Although the reason for the increase in the proportion of drift cannot be fully explained, combined with the results of previous studies, it is speculated that the increase in the relative importance of drift in this study may be related to the degradation of antibiotics. Section 4.1 has already analyzed the promoting effect of the warming environment on antibiotic degradation, which leads to lower pollution stress in the soil bacterial community of the In treatment, which is similar to the conclusion of Zhang et al. [12]. Analysis of the top 10 bins (ranked by the relative abundance of each bin) that underwent ecological process changes due to warming showed that the dominant bacteria in four bins (*Nocardioides*, *g_unclassified_Actinobacteria, Iamia*, and *AKYG1722*) shifted from a high proportion of dispersal limitation to a high proportion of drift, and one bin (*Gitt-GS-136*) shifted from Homogeneous selection to drift (Table S5). Interestingly, these dominant bacteria (*Nocardioides*, *Iamia*, *Gitt-GS-136*, and *AKYG1722*) are often involved in the decomposition of soil organic matter, pollutant degradation, and soil element cycling, and this functional enrichment helps to enhance the ability of the belonging bacterial groups to resist antibiotic pollution stress [39,40,41,42]. Overall, the warming process can enhance the dispersal of bacterial communities and promote community succession through drift.

The differences in ecological processes between the two treatments are similar to the performance of co-occurrence networks. Although the proportion of positive connections between edges in the two networks is not significantly different, the differences in the number of nodes and edges in the two networks indicate that the Outside treatment has complex biological community information transmission and connections between microbial species (Figure 6a,b), and five characteristic modules are formed. Except for the bacteria contained in M11, which disappeared due to the initial destructive tillage, the ASVs contained in other modules have the characteristic of rapid reproduction at specific soil temperatures. Although the seven characteristic modules in the In treatment also have the above characteristics, three of them belong to the dominant bacterial groups at T8 (M10, M27, M31), and M17, which is similar to the function of M11 in the Outside network, contains fewer ASVs in both abundant and rare categories than M11, indicating that the greenhouse environment improves the survival ability of the bacterial group and gradually evolves the original psychrophilic bacteria into mesophilic bacteria. The emergence of the M2 module also indicates that long-term environmental differences lead to the emergence of new characteristic communities in the soil, which may be one of the reasons for the increase in the proportion of drift in the In treatment. Key taxa identified from the module hubs and connectors of the community network play a key role in network connections. The differences in the number of key taxa in the two groups also correspond to the number of modules in the network. The In treatment has a small range and a large number of modules (a total of 75 modules), while the Outside treatment has a large range and a small number of modules (a total of 21 modules). This difference indicates that the disturbance of the open environment promotes close connections between communities in the Outside, while the bacterial groups in the In treatment are more independent.

In addition, the total relative abundance of key taxa in both groups was relatively low, and the proportion of rare taxa was 62.3% (In) and 71.9% (Outside), respectively (Table S8). This high proportion indicates that rare taxa play a dominant role in the construction and connection of microbial communities, while the In treatment increased the number of abundant taxa. Further analysis of the 20 representative taxa that were screened based on the random forest model according to feature importance showed that the high proportion of rare taxa also indicates their importance in the communication or competition process of soil communities (Tables S9 and S10). Combined with the Spearman analysis of residual antibiotic concentrations, it was found that some taxa showed specific correlations (Figure 7c, Tables S9 and S10).

For instance, in the In treatment, *JG30-KF-AS9* (ASV_86559), an antibiotic-resistant bacterium belonging to the Ktedonobacteria, was found to possess the ability to degrade organic pollutants in soil [12,43,44]. Additionally, cold-tolerant *Sporosarcina* (ASV_51196) [45]; *Geodermatophilus* (ASV_23557), capable of producing extremozymes to adapt to extreme conditions [46]; and *Gemmatimonas* (ASV_106588), involved in phosphorus metabolism, were identified [47], indicating their crucial roles in adapting to harsh environments and participating in material cycling. Furthermore, *g_unclassified_Micrococcaceae* (ASV_30639) and *g_unclassified_Intrasporangiaceae* (ASV_21711) were present in the In group, suggesting their potential ability to degrade or tolerate pollutants. Notably, Intrasporangiaceae, as an abundant family, exhibited higher abundance during soil freeze–thaw cycles, indicating its cold tolerance. In the Outside treatment, *Lysobacter* (ASV_845) and *Conexibacter* (ASV_30438) demonstrated soil remediation capabilities, respectively, degrading organic pollutants and lincomycin [48,49,50]; along with *Subgroup_7* (ASV_53853), associated with heavy metal pollution [51,52]; and *Sericytochromatia* (ASV_12533), positively correlated with the production of bacterial extracellular polymeric substances [44]. These findings suggested that these taxa played significant roles in enhancing soil remediation functions and promoting the survival of soil microorganisms. However, the functions of *67-14* (ASV_50216) and *g__unclassified_Rhizobiaceae* (ASV_36013) in contaminated soil still require further investigation.

Overall, soil microbial communities subjected to different environmental conditions for extended periods exhibited significant changes in their connectivity, soil remediation capabilities, and functions related to alleviating environmental stress. The correlation analysis with residual antibiotic concentrations indicated that the aforementioned microbial taxa were susceptible to variations in antibiotic concentrations. Additionally, except for Intrasporangiaceae, other microbial taxa exhibiting significant functions were predominantly rare taxa, further indicating that rare taxa are not only essential components of microbial communities but also play diverse ecological roles in the assembly and maintenance of microbial communities, as similarly concluded in the study by Wang et al. [17].

It is noteworthy that among the microbial taxa that did not exhibit significant correlations with antibiotic concentrations, the number of abundant taxa was higher in the In treatment compared to the Outside treatment (Tables S9 and S10). For instance, *Flavisolibacter* (ASV_82598), *Nocardioides* (ASV_44014), and *B10-SB3A* (ASV_40503) were capable of remediating heavy metals, degrading organic pollutants, and decomposing carbohydrates, respectively [40,53,54], while *Gemmatimonas* (ASV_83083) and *A4b* (ASV_57264) were involved in soil element metabolism [47,55]. In contrast, the functional taxa in the Outside treatment were *Devosia* (ASV_9950), a mycotoxin-degrading bacterium [56], and *AKYG1722* (ASV_57024), a heavy-metal-remediating bacterium [57]. This indicated that the In treatment harbored a more diverse community of functional microorganisms that were unaffected by antibiotics. Although the Outside treatment exhibited a higher abundance of microorganisms with pollution remediation functions based on correlation analysis, considering the relatively narrow ecological niches of rare taxa, their contributions to the overall soil ecosystem might be limited. Furthermore, combined with the observation that warming promotes the succession of rare taxa to abundant taxa, it can be inferred that the succession of microbial communities with degradation functions is more stable in warming environments, which may explain the higher efficiency of antibiotic degradation in warmed soils compared to non-warmed soils.

## 5. Conclusions

This study investigated the effects of greenhouse warming on the structure, function, and antibiotic degradation of soil microbial communities. The results of this study demonstrated that greenhouse warming effectively shortened the soil freezing period in northern regions, significantly reducing the residual concentrations of tetracyclines in agricultural soils, especially during winter months (T1–T5). Compared with unwarmed soils, the degradation rates of oxytetracycline, doxycycline, and chlortetracycline were significantly higher in warmed soils, with increases of 20.0%, 25.3%, and 21.3%, respectively. Although warming also promoted the overall degradation of fluoroquinolones, the differences in degradation rates among treatments were less pronounced in winter compared to tetracyclines. The semi-closed environment created by the greenhouse reduced the impact of external disturbances on soil microbial communities, promoting the positive growth of the most abundant taxa and the emergence of new characteristic communities. This shift in community structure may be related to the transition of bacterial community assembly processes from dispersal limitation to drift caused by warming, increasing the randomness of community succession. Additionally, long-term warming promoted the succession of the community towards thermophilic bacterial communities and decreased community aggregation. Analysis of microbial community networks revealed that rare taxa played a significant role in community dynamics and had potential roles in bioremediation, such as *JG30-KF-AS9* in warmed soil and *Lysobacter* and *Conexibacter* in unwarmed soil. Nevertheless, warming also increased the relative abundance of degradation functional taxa unaffected by antibiotics, such as *Nocardioides* and *B10-SB3A*, suggesting that warming may enhance the overall degradation capacity of soil by increasing the diversity of degradation pathways. Future studies should focus on identifying specific microbial taxa and associated enzymes involved in antibiotic degradation in cold environments to further elucidate the microbial mechanisms of antibiotic degradation.

## Figures and Tables

**Table 1:** The total degradation rates of six antibiotics in In and Outside treatments during the observation period.

Tetracyclines	Mean ± SD (%)		Fluoroquinolones	Mean ± SD (%)	
	In	Outside		In	Outside
Oxytetracycline	60.7 ± 1.0	50.6 ± 0.4	Norfloxacin	67.0 ± 1.5	56.7 ± 1.9
Chlortetracycline	83.4 ± 0.6	69.0 ± 0.6	Enrofloxacin	59.0 ± 1.2	41.9 ± 1.7
Doxycycline	76.8 ± 1.0	58.8 ± 0.2	Ciprofloxacin	67.6 ± 1.4	51.1 ± 1.4

**Table 2:** The number of bins and total relative abundance of the dominant ecological process in In and Outside treatments.

Ecological Process	Heterogeneous Selection (%)	Homogeneous Selection (%)	Dispersal Limitation (%)	Homogenizing Dispersal (%)	Drift (%)
Outside	0 (0%) ^a^	19 (26.3%)	56 (27.9%)	0 (0%)	46 (45.8%)
In	2 (1.5%)	18 (31.1%)	30 (9.8%)	2 (3.1%)	69 (54.5%)

^a^ a (b): number of bins (total relative abundance).

## Data Availability

Data will be made available on request.

## Supplementary Materials

The following supporting information can be downloaded at https://www.mdpi.com/article/10.3390/toxics12090667/s1: Text S1: The Preparation of Antibiotic Extraction Solution and the Activation of Solid Phase Extraction Column [18,58]; Text S2: The Quantitative Conditions and Recovery Rates for Antibiotics; Table S1: Antibiotic Recovery Rates in Soil; Table S2: The degradation rate gaps between the two treatments for each of the six antibiotics; Table S3: Top 10 abundant and rare phyla and families; Table S4: Relative importance of different ecological processes for abundant and rare bacterial communities in In and Outside treatments at four time points; Table S5: Relative abundance, relative importance of dominant ecological processes, and biological classification of corresponding enriched taxa for 58 bins; Table S6: Taxa identified in the Outside treatment using Zi-Pi screening; Table S7: Taxa identified in the In treatment using Zi-Pi screening; Table S8: Number, proportion, and mean abundance of abundant and rare ASVs in the two treatment groups, as determined by Zi-Pi screening; Table S9: Top 20 key ASVs and their associated information identified by random forest analysis in the In treatment; Table S10: Top 20 key ASVs and their associated information identified by random forest analysis in the Outside treatment; Figure S1: Degradation rate of antibiotics under two treatments during the experiment; Figure S2: Differences in the relative importance of different ecological processes for the 121 bins between the two treatments.
