# Supplementary material for: Impact of Temperature Elevation on Microbial Communities and Antibiotic Degradation in Cold Region Soils of Northeast China
Source: Toxics. 2024 Sep 13;12(9):667. doi: 10.3390/toxics12090667 (PMC11436246; doi:10.3390/toxics12090667)
Supplement: Supplementary file 1 [file toxics-12-00667-s001.zip › toxics-3161216-SI.pdf]

Supplementary materials

Impact of Temperature Elevation on Microbial Communities and Antibiotic Degradation in Cold Region Soils of Northeast China

Zijun Ni [1, 2], Xiaorong Zhang [1, 3], Shuhai Guo [3], Huaqi Pan [4, *], Zongqiang Gong [1, 3, **]

1 Key Laboratory of Pollution Ecology and Environmental Engineering, Institute of Applied Ecology, Chinese Academy of Sciences, Shenyang 110016, PR China

2 University of Chinese Academy of Sciences, Beijing 100049, PR China

3 National-Local Joint Engineering Laboratory of Contaminated Soil Remediation by Bio-physicochemical Synergistic Process, Institute of Applied Ecology, Chinese Academy of Sciences, Shenyang, 110016, PR China

4 CAS Key Laboratory of Forest Ecology and Silviculture, Institute of Applied Ecology, Chinese Academy of Sciences, Shenyang 110016, PR China

[*] Corresponding author.
[**] Corresponding author.
E-mail addresses: panhq@iae.ac.cn (H. Pan), zgong@iae.ac.cn (Z. Gong)

Text S1 The Preparation of Antibiotic Extraction Solution and the Activation of Solid Phase Extraction Column

Composite Antibiotic Standard Stock Solution: Precisely weigh 0.0200 g each of enrofloxacin, ciprofloxacin, norfloxacin, oxytetracycline, chlortetracycline and doxycycline (all reagent grade, ≥98.0%, supplied by Biotopped), and dissolve each in 10 mL of acetonitrile:water=1:1(v/v) using ultrasonication. The solutions are then brought to volume in a 25 mL volumetric flask to create a concentration of 800 mg/L for each antibiotic standard stock solution. Take 10 mL of each stock solution, mix, and dilute in a 100 mL volumetric flask to prepare a concentration of 80 mg/L of the mixed antibiotic standard stock solution, which is then stored at -18°C protected from light, with a shelf life of 1 week [1]. All standard solutions used in experiments, ranging from 0.005 to 1 mg/L, are diluted using methanol containing 1% formic acid.

Na_2EDTA-Mcllvaine Buffer Solution: Mix 1000 mL of 0.1 mol/L citric acid solution with 625 mL of 0.2 mol/L disodium hydrogen phosphate solution, adjusting the pH to 4.0 using either hydrochloric acid or sodium hydroxide. Then, add 60.5 g of Na_2EDTA to 1625 mL of Mcllvaine buffer solution and dissolve using ultrasonication to produce the Na_2EDTA-Mcllvaine buffer solution [1].

Activation of SAX-HLB Tandem Solid Phase Extraction Columns: Sequentially rinse the SAX-HLB tandem solid phase extraction columns (combination of strong anion exchange cartridge from Biotage, Charlotte, NC, USA, and hydrophilic lipophilic balance cartridges, 6 mL, 200 mg, purchased from Waters, Milford, MA, USA) with 6 mL methanol, 6 mL ultrapure water, and 6 mL disodium hydrogen phosphate solution [2].

Text S2 The Quantitative Conditions and Recovery Rates for Antibiotics

Antibiotic Quantification:

Chromatographic Conditions: Separation is performed on an Agilent Eclipse Plus C18 column (1.9 μm, 150 mm x 2.1 mm) at a column temperature of 40°C and an equilibration time of 30 min.

The flow rate is set at 0.4 mL/min with an injection volume of 10 μL. The mobile phase consists of eluent A (5 mM ammonium acetate in 0.1% formic acid) and eluent B (acetonitrile). The antibiotics are separated using a gradient program: 0~0.5 min: 98% A, 0.5~3 min: 98%~2% A, 3~5 min: 2% A, 5~8 min: 2%~98% A.

Mass Spectrometry Conditions: Employ a triple quadrupole mass spectrometer with an electrospray ionization (ESI) source and multiple reaction monitoring (MRM) mode. The auxiliary gas is set at 55 mL/min, spray voltage at 5500 V, curtain gas at 30 mL/min, and vaporizer temperature at 550°C, with collision energies of 20/27 V and cone voltage of 45 V.

Antibiotic Recovery Rates:

The calibration curve within the concentration range of 0.005 to 0.5 mg/L has an $R^2 > 0.99$. Recovery rates are determined by spiking actual samples and are as listed in Table S1, with a relative standard deviation (RSD) less than 8.6%.

references:

1. Guo, L.; Chen, Y.Q.; Zhang, L.Y.; Yang, W.J.; He, P.L. Development and Validation of a Liquid Chromatographic/Tandem Mass Spectrometric Method for Determination of Chlortetracycline, Oxytetracycline, Tetracycline, and Doxycycline in Animal Feeds. *J. AOAC Int.* **2012**, *95*, 1010-1015, http://doi.org/10.5740/jaoacint.11-087.
2. Patyra, E.; Kwiatek, K.; Nebot, C.; Gavilán, R.E. Quantification of Veterinary Antibiotics in Pig and Poultry Feces and Liquid Manure as a Non-Invasive Method to Monitor Antibiotic Usage in Livestock by Liquid Chromatography Mass-Spectrometry. *Molecules* **2020**, *25*, 25143265, http://doi.org/10.3390/molecules25143265.

Table S1. Antibiotic Recovery Rates in Soil

Types	Antibiotics	0.005-0.5 mg/kg Recovery (%)
Tetracyclines	Doxycycline	80.8-101.8
	Oxytetracycline	72.6-90.3
	Chlortetracycline	67.1-78.3
Fluoroquinolones	Enrofloxacin	42.5-49.8
	Norfloxacin	45.2-50.1
	Ciprofloxacin	53.1-56.9

Table S2. The degradation rate gaps between the two treatments for each of the six antibiotics.

	Time	T1	T2	T3	T4	T5	T6	T7	T8	T9	T10	T11	T12
Degradation rate gap (%)	Oxytetracycline	5.2	13.3	18	20	18.9	14.3	11.5	11.6	10.3	8.9	9.1	10.1
	Doxycycline	8.6	14.4	18.9	25	25.3	20.9	19.7	19.9	18.6	18.7	19.1	18
	Chlortetracycline	10.1	13.4	21.3	20.9	16.3	13.4	11.8	9.5	9.9	10.9	12.9	14.4
	Enrofloxacin	-0.4	1.8	2.1	5.1	6.4	6.8	8	10.2	12.3	13.5	15.4	17.1
	Ciprofloxacin	-1.4	-2.6	-2.7	-1.7	3.8	9	12.9	12.7	14	15.5	16.8	16.4
	Norfloxacin	1.2	5.2	4.3	7	5.9	4.3	6.1	8.1	7.5	8.3	8.8	10.3

Table S3. Top 10 abundant and rare phyla and families.

	Outside-T0	Outside-T4	Outside-T8	Outside-T12	In-T0	In-T4	In-T8	In-T12
Relative abundance of abundant phylum-level taxa (%)								
Actinobacteriota	22.76	31.85	26.49	20.76	31.36	38.15	23.06	38.54
Proteobacteria	14.97	23.63	20.30	26.63	18.44	16.83	22.61	17.90
Chloroflexi	22.49	9.05	14.15	9.98	11.11	12.07	13.02	9.63
Gemmatimonadota	6.20	5.16	5.27	9.38	7.61	6.63	7.17	4.72
Acidobacteriota	3.15	2.09	3.90	2.49	3.29	2.78	4.93	1.20
Firmicutes	0.53	1.95	1.37	1.64	0.72	0.70	1.30	1.96
Bacteroidota	2.03	1.66	0.59	1.04	0.83	0.76	0.84	0.20
Myxococcota	1.55	0.67	0.62	0.58	0.80	0.59	0.64	0.46
Patescibacteria	0.72	0.03	0.00	0.00	0.32	0.28	0.51	1.19
Nitrospirota	0.26	0.16	0.11	0.24	0.41	0.26	0.62	0.76
Others	1.42	0.50	0.88	0.40	1.01	0.88	1.41	0.62
Relative abundance of rare phylum-level taxa (%)								
Proteobacteria	5.69	6.99	7.71	9.10	6.77	5.25	6.97	5.75
Actinobacteriota	4.72	6.71	5.74	4.78	5.48	5.80	3.95	6.68
Chloroflexi	2.25	1.05	1.87	1.85	2.17	2.34	2.43	2.01
Firmicutes	1.69	2.62	1.47	1.26	1.69	1.25	1.50	2.48
Bacteroidota	1.86	1.89	1.74	2.90	1.77	1.18	1.41	0.87
Acidobacteriota	1.03	0.87	2.80	1.85	1.19	0.93	2.29	1.22
Gemmatimonadota	1.41	0.68	1.62	1.44	1.43	1.10	1.42	1.28
Myxococcota	1.52	0.67	1.30	1.18	0.91	0.57	1.19	0.55
Verrucomicrobiota	1.13	0.47	0.42	0.68	0.76	0.31	0.50	0.37
Patescibacteria	0.61	0.33	0.39	0.44	0.41	0.33	0.71	0.45
Others	2.01	0.98	1.28	1.37	1.51	1.01	1.52	1.16
Relative abundance of abundant family-level taxa (%)								

Gemmatimonadaceae	5.41	5.04	5.08	9.36	7.51	6.50	6.95	4.56
Micrococcaceae	0.40	7.99	4.86	4.91	7.79	7.39	1.92	8.93
Nocardioidaceae	1.48	6.00	3.05	1.91	4.55	6.25	3.79	4.90
Intrasporangiaceae	0.20	5.13	0.90	0.90	4.83	7.94	1.20	3.69
Sphingomonadaceae	1.11	2.62	4.91	1.79	2.98	4.39	3.17	2.19
Microbacteriaceae	2.80	2.71	2.54	1.93	1.44	1.74	2.22	2.03
Xanthobacteraceae	1.00	1.66	2.03	2.66	2.06	1.46	2.37	1.49
Rhodanobacteraceae	1.86	1.59	1.17	2.52	0.86	0.94	1.53	1.73
Solirubrobacteraceae	2.08	0.99	1.19	1.54	0.98	1.10	0.73	1.73
Mycobacteriaceae	1.50	1.44	1.38	1.27	1.09	1.12	0.96	1.53
Relative abundance of rare family-level taxa (%)								
Gemmatimonadaceae	1.11	0.46	1.28	1.26	1.15	0.86	1.16	1.00
Nocardioidaceae	0.62	1.52	0.52	0.55	0.81	0.93	0.24	0.52
Comamonadaceae	0.64	0.93	0.92	1.06	1.00	0.59	0.37	0.11
Chitinophagaceae	0.69	0.82	0.78	1.04	0.56	0.51	0.48	0.56
Xanthomonadaceae	0.34	0.76	0.94	0.48	0.57	0.58	0.41	0.15
Bacillaceae	0.38	0.71	0.52	0.38	0.41	0.42	0.52	0.80
Solirubrobacteraceae	0.50	0.34	0.40	0.43	0.42	0.41	0.27	0.61
Micrococcaceae	0.07	0.81	0.31	0.21	0.42	0.45	0.15	0.92
Oxalobacteraceae	0.21	1.27	0.40	0.78	0.20	0.15	0.14	0.13
Microbacteriaceae	0.39	0.83	0.35	0.50	0.39	0.25	0.20	0.36

Table S4. Relative importance of different ecological processes for abundant and rare bacterial communities in In and Outside treatments at four time points.

Ecological process		Heterogeneous selection (%)	Homogeneous selection (%)	Dispersal limitation (%)	Homogenezing dispersal (%)	Drift (%)
Abbreviation		HeS	HoS	DL	HD	DR
Abundant	Outside	0.9	30.6	1.0	25.7	41.7
		0.2	41.5	2.2	32.6	23.5
		0.3	29.9	4.0	25.7	40.0
		0.0	46.3	2.5	27.4	23.7
	In	0.0	40.5	1.3	18.4	39.8
		0.4	39.6	0.2	34.0	25.8
		1.6	29.8	2.5	28.1	38.0
		0.0	36.2	3.6	34.9	25.4
Rare	Outside	2.9	14.0	10.1	22.9	50.1
		1.1	22.3	10.1	21.5	45.0
		0.8	15.4	12.0	15.9	55.9
		0.5	24.4	19.1	16.0	40.0
	In	0.0	21.6	12.5	10.7	55.2
		0.8	20.6	4.1	21.3	53.2
		1.3	20.2	12.3	10.1	56.0
		0.1	25.9	10.3	21.8	41.9

Table S5. Relative abundance, relative importance of dominant ecological processes, and biological classification of corresponding enriched taxa for 58 bins.

Bin	Top Taxon ID	Total RA in Bin (%)		Phylum	Class	Order	Family	Genus	Ecological process		Percentage(%)	
		Outside	In						Outside	In	Outside	In
Bin3	ASV_58193	3.6	5.0	Actinobacteriota	Actinobacteria	Propionibacteriales	Nocardioidaceae	Nocardioides	DL	DR	45.6	76.6
Bin33	ASV_52702	4.4	2.6	Chloroflexi	Gitt-GS-136	Gitt-GS-136	Gitt-GS-136	Gitt-GS-136	HoS	DR	62.0	62.8
Bin89	ASV_9950	3.6	2.6	Proteobacteria	Alphaproteobacteria	Rhizobiales	Devosiaceae	Devosia	DR	HoS	52.4	95.9
Bin107	ASV_75855	2.1	4.0	Actinobacteriota	Actinobacteria	Micrococcales	Micrococcaceae	Arthrobacter	DR	HoS	64.8	99.5
Bin52	ASV_90686	2.4	1.8	Chloroflexi	Chloroflexia	Thermomicrobiales	JG30-KF-CM45	JG30-KF-CM45	DR	HD	55.1	84.4
Bin1	ASV_30090	2.6	1.5	Actinobacteriota	Actinobacteria	Corynebacteriales	Mycobacteriaceae	Mycobacterium	DR	HoS	62.3	77.8
Bin2	ASV_50439	2.5	1.6	Actinobacteriota	Actinobacteria	unclassified_Actinobacteria	unclassified_Actinobacteria	unclassified_Actinobacteria	DL	DR	58.1	94.3
Bin51	ASV_13528	2.3	1.4	Chloroflexi	Chloroflexia	Thermomicrobiales	AKYG1722	AKYG1722	DL	DR	61.3	63.6
Bin34	ASV_16873	1.5	1.8	Chloroflexi	KD4-96	KD4-96	KD4-96	KD4-96	DR	HoS	65.8	100.0
Bin30	ASV_63258	1.8	1.1	Actinobacteriota	Acidimicrobiia	Microtrichales	Iamiaceae	Iamia	DL	DR	61.1	72.6
Bin84	ASV_80337	1.6	1.2	Proteobacteria	Gammaproteobacteria	Xanthomonadales	Rhodanobacteraceae	Rhodanobacter	DR	HeS	62.8	47.0
Bin116	ASV_106932	0.8	1.7	Proteobacteria	Gammaproteobacteria	Burkholderiales	SC-I-84	SC-I-84	DR	HoS	57.6	100.0
Bin41	ASV_14657	0.8	1.3	Chloroflexi	OLB14	OLB14	OLB14	OLB14	HoS	DR	100.0	80.8
Bin105	ASV_106316	0.7	1.3	Actinobacteriota	Actinobacteria	Streptomycetales	Streptomycetaceae	Streptomyces	DR	DL	92.8	17.5
Bin81	ASV_16441	1.0	0.9	Chloroflexi	Chloroflexia	Thermomicrobiales	JG30-KF-CM45	JG30-KF-CM45	DR	HD	79.9	53.0
Bin94	ASV_51724	1.0	0.9	Proteobacteria	Gammaproteobacteria	Xanthomonadales	Xanthomonadaceae	Thermomonas	DL	DR	54.5	48.9
Bin35	ASV_9752	0.9	0.8	Chloroflexi	Anaerolineae	SBR1031	A4b	A4b	HoS	DR	92.4	59.7

Bin117	ASV_81727	0.8	0.9	Acidobacteriota	Vicinamibacteria	Vicinamibacterales	uncultured	uncultured	DR	DL	54.9	34.8
Bin118	ASV_80217	0.8	0.7	Acidobacteriota	Vicinamibacteria	Vicinamibacterales	Vicinamibacteraceae	Vicinamibacteraceae	DL	DR	61.4	79.6
Bin109	ASV_58152	1.1	0.4	Proteobacteria	Gammaproteobacteria	Burkholderiales	Oxalobacteraceae	Massilia	DL	DR	54.5	59.0
Bin5	ASV_9703	0.7	0.7	Actinobacteriota	Actinobacteria	Propionibacteriales	Nocardioidaceae	Nocardioides	DL	DR	53.6	71.8
Bin95	ASV_6904	0.7	0.7	Proteobacteria	Gammaproteobacteria	Xanthomonadales	Xanthomonadaceae	Luteimonas	DR	HoS	91.2	94.1
Bin114	ASV_69468	1.0	0.4	Proteobacteria	Gammaproteobacteria	Burkholderiales	Comamonadaceae	Rhizobacter	DL	DR	75.0	55.8
Bin74	ASV_23242	0.7	0.6	Proteobacteria	Gammaproteobacteria	Gammaproteobacteria_Incertae_Sedis	Unknown_Family	Acidibacter	HoS	DR	47.6	91.8
Bin17	ASV_73340	0.7	0.5	Myxococcota	Polyangia	Haliangiales	Haliangiaceae	Haliangium	DL	DR	57.5	82.3
Bin83	ASV_8607	0.6	0.5	Proteobacteria	Alphaproteobacteria	Micropepsales	Micropepsaceae	uncultured	DL	DR	57.8	50.7
Bin10	ASV_31111	0.3	0.8	Actinobacteriota	Acidimicrobiia	IMCC26256	IMCC26256	IMCC26256	DR	HoS	45.2	61.3
Bin56	ASV_14431	0.4	0.7	Firmicutes	Bacilli	Bacillales	Bacillaceae	Bacillus	DR	DL	58.8	48.9
Bin119	ASV_48215	0.7	0.3	Acidobacteriota	Thermoanaerobaculia	Thermoanaerobaculales	Thermoanaerobaculaceae	Subgroup_10	DL	DR	85.3	90.3
Bin61	ASV_61886	0.6	0.4	Deinococcota	Deinococci	Deinococcales	Trueperaceae	Truepera	DL	DR	53.2	89.4
Bin46	ASV_103479	0.7	0.3	Bacteroidota	Bacteroidia	Chitinophagales	Chitinophagaceae	uncultured	DL	DR	82.5	83.6
Bin98	ASV_84234	0.3	0.6	Proteobacteria	Gammaproteobacteria	Pseudomonadales	Pseudomonadaceae	Pseudomonas	DR	DL	45.2	17.0
Bin38	ASV_79023	0.4	0.5	Chloroflexi	Anaerolineae	SBR1031	A4b	A4b	DL	DR	52.5	75.5
Bin58	ASV_70003	0.7	0.2	Firmicutes	Bacilli	Bacillales	Planococcaceae	Sporosarcina	DL	DR	79.5	80.8
Bin40	ASV_63108	0.5	0.3	Chloroflexi	Chloroflexia	Thermomicrobiales	Thermomicrobiaceae	Nitrolancea	HoS	DR	39.6	82.3
Bin86	ASV_76238	0.4	0.4	Proteobacteria	Gammaproteobacteria	Burkholderiales	Nitrosomonadaceae	Ellin6067	HoS	DR	36.2	84.7

Bin111	ASV_50559	0.5	0.3	Proteobacteria	Gammaproteobacteria	Burkholderiales	Nitrosomonadaceae	Nitrosospira	DL	DR	52.9	95.5
Bin121	ASV_44716	0.5	0.2	Proteobacteria	Gammaproteobacteria	Burkholderiales	Comamonadaceae	unclassified_Comamonadaceae	DL	DR	76.9	42.6
Bin91	ASV_61224	0.5	0.2	Proteobacteria	Alphaproteobacteria	Rhodobacterales	Rhodobacteraceae	Amaricoccus	HoS	DR	40.0	96.7
Bin59	ASV_38317	0.5	0.2	Firmicutes	Bacilli	Bacillales	Planococcaceae	Sporosarcina	DL	DR	61.2	58.4
Bin37	ASV_60837	0.1	0.6	Chloroflexi	Ktedonobacteria	Ktedonobacterales	JG30-KF-AS9	JG30-KF-AS9	HoS	DR	74.5	74.7
Bin113	ASV_102435	0.4	0.3	Proteobacteria	Gammaproteobacteria	Burkholderiales	Nitrosomonadaceae	IS-44	DL	DR	64.7	47.1
Bin50	ASV_91588	0.4	0.3	Bacteroidota	Bacteroidia	Cytophagales	Microscillaceae	uncultured	DL	DR	59.7	71.2
Bin53	ASV_67210	0.3	0.4	Firmicutes	Clostridia	Peptostreptococcales-Tissierellales	Family_XI	Anaerosalibacter	DR	DL	83.7	35.8
Bin48	ASV_48984	0.4	0.3	Bacteroidota	Bacteroidia	Chitinophagales	Chitinophagaceae	Terrimonas	DL	DR	79.8	94.7
Bin31	ASV_108992	0.4	0.2	Actinobacteriota	Acidimicrobiia	Microtrichales	Ilumatobacteraceae	CL500-29_marine_group	DL	DR	60.7	99.0
Bin71	ASV_25713	0.3	0.3	Gemmatimonadota	S0134_terrestrial_group	S0134_terrestrial_group	S0134_terrestrial_group	S0134_terrestrial_group	DL	DR	50.8	96.6
Bin68	ASV_39847	0.3	0.2	Gemmatimonadota	Gemmatimonadetes	Gemmatimonadales	Gemmatimonadaceae	uncultured	DL	DR	68.2	93.7
Bin23	ASV_65638	0.3	0.2	Myxococcota	Polyangia	Polyangiales	BIrii41	BIrii41	DL	DR	90.0	49.4
Bin32	ASV_75958	0.2	0.2	Acidobacteriota	Acidobacteriae	Solibacterales	Solibacteraceae	Candidatus_Solibacter	DL	DR	43.5	43.0
Bin24	ASV_105802	0.3	0.1	Verrucomicrobiota	Verrucomicrobiae	Chthoniobacterales	Chthoniobacteraceae	Chthoniobacter	DL	DR	90.9	63.5
Bin93	ASV_36026	0.3	0.1	Proteobacteria	Alphaproteobacteria	Sphingomonadales	Sphingomonadaceae	Qipengyuania	DL	DR	62.4	91.2
Bin13	ASV_69288	0.3	0.1	Gemmatimonadota	S0134_terrestrial_group	S0134_terrestrial_group	S0134_terrestrial_group	S0134_terrestrial_group	DL	DR	57.8	98.1

Bin96	ASV_64155	0.1	0.1	Proteobacteria	Gammaproteobacteria	Xanthomonadales	Xanthomonadaceae	Pseudoxanthomonas	DL	DR	59.8	48.8
Bin87	ASV_30899	0.2	0.1	Proteobacteria	Alphaproteobacteria	uncultured	uncultured	uncultured	DL	DR	84.4	46.8
Bin64	ASV_46202	0.1	0.2	Firmicutes	Clostridia	Lachnospirales	Lachnospiraceae	uncultured	DR	DL	67.0	21.0
Bin60	ASV_90300	0.1	0.1	Dependentiae	Babeliae	Babeliales	Vermiphilaceae	Vermiphilaceae	DR	DL	73.4	34.6
Bin45	ASV_97061	0.1	0.1	Bacteroidota	Bacteroidia	Chitinophagales	Chitinophagaceae	Edaphobaculum	DL	HeS	66.2	38.0

Table S6. Taxa identified in the Outside treatment using Zi-Pi screening.

ASV	modu	Zi-Pi	Phylum	Class	Order	Family	Genus	Type
ASV_89616	15	Module hub	Bacteroidota	Bacteroidia	Sphingobacteriales	unclassified	unclassified	Abundant
ASV_77647	15	Module hub	Verrucomicrobiota	Verrucomicrobiae	Pedosphaerales	Pedosphaeraceae	Pedosphaeraceae	Abundant
ASV_44907	15	Module hub	Bacteroidota	Bacteroidia	Chitinophagales	Chitinophagaceae	uncultured	Abundant
ASV_34720	15	Module hub	Bacteroidota	Bacteroidia	Sphingobacteriales	KD3-93	KD3-93	Abundant
ASV_19668	N	Module hub	Firmicutes	Bacilli	Bacillales	Planococcaceae	Sporosarcina	Abundant
ASV_110096	15	Module hub	Chloroflexi	Chloroflexia	Thermomicrobiales	JG30-KF-CM45	JG30-KF-CM45	Abundant
ASV_102931	15	Module hub	Bacteroidota	Bacteroidia	Chitinophagales	Chitinophagaceae	Ferruginibacter	Abundant
ASV_9950	14	Connector	Proteobacteria	Alphaproteobacteria	Rhizobiales	Devosiaceae	Devosia	Abundant
ASV_98219	N	Connector	Chloroflexi	Dehalococcoidia	S085	S085	S085	Abundant
ASV_95947	15	Connector	Gemmatimonadota	Gemmatimonadetes	Gemmatimonadales	Gemmatimonadaceae	unclassified	Abundant
ASV_954	N	Connector	Proteobacteria	Alphaproteobacteria	Rhizobiales	Rhizobiales_Incertae_Sedis	Bauldia	Abundant
ASV_94713	12	Connector	Proteobacteria	Alphaproteobacteria	Rhizobiales	Hyphomicrobiaceae	Hyphomicrobium	Abundant
ASV_92521	12	Connector	Proteobacteria	Alphaproteobacteria	Reyranellales	Reyranellaceae	Reyranella	Abundant
ASV_89855	12	Connector	Chloroflexi	TK10	TK10	TK10	TK10	Abundant
ASV_8938	N	Connector	Chloroflexi	Chloroflexia	Thermomicrobiales	Thermomicrobiaceae	Sphaerobacter	Abundant
ASV_84797	12	Connector	Actinobacteriota	Actinobacteria	Frankiales	uncultured	uncultured	Abundant
ASV_81887	14	Connector	Proteobacteria	Alphaproteobacteria	Rhizobiales	Rhizobiaceae	unclassified_Rhizobiaceae	Abundant
ASV_80217	21	Connector	Acidobacteriota	Vicinamibacteria	Vicinamibacterales	Vicinamibacteraceae	Vicinamibacteraceae	Abundant

ASV_79421	21	Connector	Proteobacteria	Gammaproteobacteria	Xanthomonadales	Xanthomonadaceae	unclassified	Abundant
ASV_7854	15	Connector	Actinobacteriota	Actinobacteria	Micrococcales	Microbacteriaceae	unclassified	Abundant
ASV_77719	14	Connector	Actinobacteriota	Actinobacteria	Corynebacteriales	Nocardiaceae	Rhodococcus	Abundant
ASV_76433	14	Connector	Chloroflexi	Chloroflexia	Thermomicrobiales	Thermomicrobiaceae	Nitrolancea	Abundant
ASV_74377	14	Connector	Proteobacteria	Alphaproteobacteria	Rhizobiales	Xanthobacteraceae	Bradyrhizobium	Abundant
ASV_6904	15	Connector	Proteobacteria	Gammaproteobacteria	Xanthomonadales	Xanthomonadaceae	Luteimonas	Abundant
ASV_67442	21	Connector	Proteobacteria	Alphaproteobacteria	Rhizobiales	unclassified_Rhizobiales	unclassified_Rhizobiales	Abundant
ASV_64905	15	Connector	Nitrospirota	Nitrospiria	Nitrospirales	Nitrospiraceae	Nitrospira	Abundant
ASV_64853	N	Connector	Actinobacteriota	Actinobacteria	Micromonosporales	Micromonosporaceae	unclassified	Abundant
ASV_61379	12	Connector	Acidobacteriota	Thermoanaerobaculia	Thermoanaerobaculales	Thermoanaerobaculaceae	Subgroup_10	Abundant
ASV_6110	14	Connector	Actinobacteriota	Thermoleophilia	Solirubrobacterales	Solirubrobacteraceae	JCM_18997	Abundant
ASV_57024	21	Connector	Chloroflexi	Chloroflexia	Thermomicrobiales	AKYG1722	AKYG1722	Abundant
ASV_5488	21	Connector	Myxococcota	Polyangia	Nannocystales	Nannocystaceae	Nannocystis	Abundant
ASV_54758	14	Connector	Myxococcota	Polyangia	Polyangiales	BIrii41	BIrii41	Abundant
ASV_53910	12	Connector	Actinobacteriota	Thermoleophilia	Solirubrobacterales	Solirubrobacteraceae	JCM_18997	Abundant
ASV_50345	12	Connector	Gemmatimonadota	Gemmatimonadetes	Gemmatimonadales	Gemmatimonadaceae	uncultured	Abundant
ASV_48822	11	Connector	Chloroflexi	Gitt-GS-136	Gitt-GS-136	Gitt-GS-136	Gitt-GS-136	Abundant
ASV_45577	14	Connector	Actinobacteriota	Actinobacteria	Micromonosporales	Micromonosporaceae	Micromonospora	Abundant
ASV_45241	15	Connector	Acidobacteriota	Blastocatellia	Blastocatellales	Blastocatellaceae	uncultured	Abundant
ASV_43926	14	Connector	Proteobacteria	Alphaproteobacteria	Sphingomonadales	Sphingomonadaceae	Sphingomonas	Abundant
ASV_43920	12	Connector	Actinobacteriota	Actinobacteria	Kineosporiales	Kineosporiaceae	Angustibacter	Abundant
ASV_42581	15	Connector	Chloroflexi	Chloroflexia	Thermomicrobiales	JG30-KF-CM45	JG30-KF-CM45	Abundant
ASV_40780	21	Connector	Actinobacteriota	Actinobacteria	Micromonosporales	Micromonosporaceae	unclassified	Abundant
ASV_39206	15	Connector	Proteobacteria	Gammaproteobacteria	Xanthomonadales	Rhodanobacteraceae	Rhodanobacter	Abundant
ASV_38705	15	Connector	Bacteroidota	Bacteroidia	Flavobacteriales	Flavobacteriaceae	Flavobacterium	Abundant
ASV_36831	15	Connector	Actinobacteriota	Actinobacteria	Propionibacteriales	Nocardioidaceae	Nocardioides	Abundant
ASV_33810	14	Connector	Proteobacteria	Gammaproteobacteria	Burkholderiales	Oxalobacteraceae	Noviherbaspirillum	Abundant
ASV_32462	21	Connector	Proteobacteria	Alphaproteobacteria	Rhizobiales	Xanthobacteraceae	Pseudolabrys	Abundant
ASV_32379	11	Connector	Actinobacteriota	Actinobacteria	Micrococcales	Micrococcaceae	Paenarthrobacter	Abundant

ASV_31111	N	Connector	Actinobacteriota	Acidimicrobiia	IMCC26256	IMCC26256	IMCC26256	Abundant
ASV_29973	14	Connector	Actinobacteriota	Actinobacteria	unclassified	unclassified	unclassified	Abundant
ASV_26031	12	Connector	Chloroflexi	KD4-96	KD4-96	KD4-96	KD4-96	Abundant
ASV_25774	12	Connector	Actinobacteriota	Actinobacteria	Micrococcales	Microbacteriaceae	unclassified_Microbacteriaceae	Abundant
ASV_20404	14	Connector	Proteobacteria	Alphaproteobacteria	Sphingomonadales	Sphingomonadaceae	Sphingomonas	Abundant
ASV_2009	14	Connector	Proteobacteria	Alphaproteobacteria	Rhizobiales	Xanthobacteraceae	unclassified_Xanthobacteraceae	Abundant
ASV_18445	11	Connector	Chloroflexi	Chloroflexia	Kallotenuales	AKIW781	AKIW781	Abundant
ASV_14801	14	Connector	Chloroflexi	Gitt-GS-136	Gitt-GS-136	Gitt-GS-136	Gitt-GS-136	Abundant
ASV_14239	15	Connector	Actinobacteriota	Thermoleophilia	Solirubrobacterales	67-14	67-14	Abundant
ASV_106038	14	Connector	Actinobacteriota	Thermoleophilia	Solirubrobacterales	Solirubrobacteraceae	JCM_18997	Abundant
ASV_102609	14	Connector	Myxococcota	Polyangia	Haliangiales	Haliangiaceae	Haliangium	Abundant
ASV_10129	N	Connector	Chloroflexi	Chloroflexia	Thermomicrobiales	JG30-KF-CM45	JG30-KF-CM45	Abundant
ASV_55800	15	Module hub	Actinobacteriota	Actinobacteria	Propionibacteriales	Nocardioidaceae	Nocardioides	Rare
ASV_13064	15	Module hub	Actinobacteriota	Thermoleophilia	Solirubrobacterales	Solirubrobacteraceae	JCM_18997	Rare
ASV_105603	15	Module hub	Gemmatimonadota	Gemmatimonadetes	Gemmatimonadales	Gemmatimonadaceae	Gemmatimonas	Rare
ASV_99978	21	Connector	Firmicutes	Bacilli	Bacillales	Bacillaceae	Bacillus	Rare
ASV_99864	15	Connector	Chloroflexi	TK10	TK10	TK10	TK10	Rare
ASV_99025	14	Connector	Bacteroidota	Bacteroidia	Flavobacteriales	Flavobacteriaceae	Flavobacterium	Rare
ASV_96837	N	Connector	Chloroflexi	Anaerolineae	Caldilineales	Caldilineaceae	uncultured	Rare
ASV_96659	21	Connector	Actinobacteriota	Actinobacteria	Propionibacteriales	Nocardioidaceae	Marmoricola	Rare
ASV_96527	15	Connector	Proteobacteria	Alphaproteobacteria	Defluviicoccales	uncultured	uncultured	Rare
ASV_95858	15	Connector	Bacteroidota	Bacteroidia	Chitinophagales	Chitinophagaceae	Segetibacter	Rare
ASV_95322	N	Connector	Proteobacteria	Alphaproteobacteria	uncultured	uncultured	uncultured	Rare
ASV_95060	14	Connector	Myxococcota	Polyangia	Polyangiales	Sandaracinaceae	uncultured	Rare
ASV_94421	14	Connector	Myxococcota	Polyangia	Polyangiales	BIrii41	BIrii41	Rare
ASV_91861	14	Connector	Proteobacteria	Gammaproteobacteria	Pseudomonadales	Cellvibrionaceae	Cellvibrio	Rare
ASV_91754	14	Connector	Acidobacteriota	Vicinamibacteria	Vicinamibacterales	uncultured	uncultured	Rare
ASV_91672	14	Connector	Firmicutes	Bacilli	Bacillales	Planococcaceae	Sporosarcina	Rare
ASV_91593	14	Connector	Firmicutes	Bacilli	Bacillales	Bacillaceae	Bacillus	Rare

ASV_90090	N	Connector	Myxococcota	Polyangia	Haliangiales	Haliangiaceae	Haliangium	Rare
ASV_9009	14	Connector	Cyanobacteria	Sericytochromatia	Sericytochromatia	Sericytochromatia	Sericytochromatia	Rare
ASV_88965	14	Connector	Firmicutes	Bacilli	Bacillales	Bacillaceae	Bacillus	Rare
ASV_88904	N	Connector	Bacteroidota	Bacteroidia	Sphingobacteriales	Sphingobacteriaceae	Pedobacter	Rare
ASV_88802	15	Connector	Chloroflexi	Chloroflexia	Chloroflexales	Herpetosiphonaceae	Herpetosiphon	Rare
ASV_88729	14	Connector	Proteobacteria	Gammaproteobacteria	Pseudomonadales	Pseudomonadaceae	Pseudomonas	Rare
ASV_88298	15	Connector	Firmicutes	Bacilli	Bacillales	Bacillaceae	Pseudogracilibacillus	Rare
ASV_87935	14	Connector	Actinobacteriota	Thermoleophilia	Gaiellales	uncultured	uncultured	Rare
ASV_87211	21	Connector	Proteobacteria	Alphaproteobacteria	Caulobacterales	Caulobacteraceae	uncultured	Rare
ASV_85297	21	Connector	Chloroflexi	Chloroflexia	Thermomicrobiales	JG30-KF-CM45	JG30-KF-CM45	Rare
ASV_845	N	Connector	Proteobacteria	Gammaproteobacteria	Xanthomonadales	Xanthomonadaceae	Lysobacter	Rare
ASV_83588	15	Connector	Chloroflexi	Chloroflexia	Thermomicrobiales	JG30-KF-CM45	JG30-KF-CM45	Rare
ASV_83542	15	Connector	Actinobacteriota	Actinobacteria	Micrococcales	Microbacteriaceae	unclassified_Microbacteriaceae	Rare
ASV_83239	14	Connector	Chloroflexi	Chloroflexia	Thermomicrobiales	JG30-KF-CM45	JG30-KF-CM45	Rare
ASV_82392	N	Connector	Proteobacteria	Gammaproteobacteria	Burkholderiales	Comamonadaceae	unclassified_Comamonadaceae	Rare
ASV_81404	14	Connector	Gemmatimonadota	Gemmatimonadetes	Gemmatimonadales	Gemmatimonadaceae	Gemmatimonas	Rare
ASV_81296	14	Connector	Bacteroidota	Bacteroidia	Flavobacteriales	Flavobacteriaceae	Flavobacterium	Rare
ASV_79758	21	Connector	Proteobacteria	Gammaproteobacteria	Pseudomonadales	Moraxellaceae	Acinetobacter	Rare
ASV_7927	15	Connector	Actinobacteriota	Acidimicrobiia	Microtrichales	Iamiaceae	Iamia	Rare
ASV_78780	N	Connector	Myxococcota	Polyangia	Haliangiales	Haliangiaceae	Haliangium	Rare
ASV_783	N	Connector	Chloroflexi	Chloroflexia	Thermomicrobiales	JG30-KF-CM45	JG30-KF-CM45	Rare
ASV_77456	21	Connector	Proteobacteria	Alphaproteobacteria	Tistrellales	Geminicoccaceae	Candidatus_Alysiosphaera	Rare
ASV_76739	15	Connector	Proteobacteria	Alphaproteobacteria	Rhizobiales	Xanthobacteraceae	uncultured	Rare
ASV_76601	14	Connector	Proteobacteria	Gammaproteobacteria	Xanthomonadales	Xanthomonadaceae	Lysobacter	Rare
ASV_73732	15	Connector	Planctomycetota	Planctomycetes	Isosphaerales	Isosphaeraceae	uncultured	Rare
ASV_72413	15	Connector	Actinobacteriota	Actinobacteria	Micrococcales	Microbacteriaceae	unclassified_Microbacteriaceae	Rare
ASV_71886	14	Connector	Actinobacteriota	Actinobacteria	Micromonosporales	Micromonosporaceae	Micromonospora	Rare
ASV_71572	14	Connector	Proteobacteria	Alphaproteobacteria	Acetobacterales	Acetobacteraceae	uncultured	Rare
ASV_71213	21	Connector	Actinobacteriota	Actinobacteria	Micromonosporales	Micromonosporaceae	unclassified	Rare

ASV_6872	14	Connector	Actinobacteriota	Actinobacteria	Streptosporangiales	Thermomonosporaceae	Actinomadura	Rare
ASV_68627	15	Connector	Acidobacteriota	Vicinamibacteria	Vicinamibacterales	uncultured	uncultured	Rare
ASV_68090	N	Connector	Bacteroidota	Bacteroidia	Sphingobacteriales	Sphingobacteriaceae	Pedobacter	Rare
ASV_66893	12	Connector	Actinobacteriota	Actinobacteria	Propionibacteriales	Nocardioidaceae	Nocardioides	Rare
ASV_66315	15	Connector	Actinobacteriota	Acidimicrobiia	Microtrichales	Iamiaceae	Iamia	Rare
ASV_66226	12	Connector	Actinobacteriota	Actinobacteria	Micromonosporales	Micromonosporaceae	unclassified	Rare
ASV_65686	11	Connector	Actinobacteriota	Thermoleophilia	Solirubrobacterales	67-14	67-14	Rare
ASV_65441	14	Connector	Proteobacteria	Alphaproteobacteria	Rhizobiales	Xanthobacteraceae	Rhodoplanes	Rare
ASV_63212	N	Connector	Planctomycetota	Phycisphaerae	Phycisphaerales	Phycisphaeraceae	SM1A02	Rare
ASV_62370	21	Connector	Firmicutes	Bacilli	Bacillales	Planococcaceae	Sporosarcina	Rare
ASV_61444	12	Connector	Bacteroidota	Bacteroidia	Cytophagales	Microscillaceae	uncultured	Rare
ASV_6040	N	Connector	Proteobacteria	Alphaproteobacteria	Rhizobiales	Rhizobiaceae	Aminobacter	Rare
ASV_5997	N	Connector	Bacteroidota	Bacteroidia	Flavobacteriales	Flavobacteriaceae	Flavobacterium	Rare
ASV_59962	N	Connector	Proteobacteria	Gammaproteobacteria	Pseudomonadales	Pseudomonadaceae	Pseudomonas	Rare
ASV_59594	14	Connector	Proteobacteria	Alphaproteobacteria	Reyranellales	Reyranellaceae	Reyranella	Rare
ASV_58886	21	Connector	Firmicutes	Bacilli	Bacillales	Planococcaceae	Sporosarcina	Rare
ASV_57777	14	Connector	Myxococcota	Polyangia	Haliangiales	Haliangiaceae	Haliangium	Rare
ASV_57690	12	Connector	Bacteroidota	Bacteroidia	Cytophagales	Microscillaceae	uncultured	Rare
ASV_56500	14	Connector	Verrucomicrobiota	Verrucomicrobiae	Opitutales	Opitutaceae	Lacunisphaera	Rare
ASV_56252	N	Connector	Proteobacteria	Gammaproteobacteria	Xanthomonadales	Rhodanobacteraceae	Rhodanobacter	Rare
ASV_55905	14	Connector	Proteobacteria	Gammaproteobacteria	Burkholderiales	SC-I-84	SC-I-84	Rare
ASV_55622	N	Connector	Acidobacteriota	Vicinamibacteria	Vicinamibacterales	Vicinamibacteraceae	Vicinamibacteraceae	Rare
ASV_54409	12	Connector	Actinobacteriota	Actinobacteria	Micromonosporales	Micromonosporaceae	unclassified	Rare
ASV_54347	15	Connector	Proteobacteria	Alphaproteobacteria	Caulobacterales	Caulobacteraceae	uncultured	Rare
ASV_54137	N	Connector	Proteobacteria	Alphaproteobacteria	Acetobacterales	Acetobacteraceae	Roseomonas	Rare
ASV_53941	14	Connector	Proteobacteria	Alphaproteobacteria	Azospirillales	Azospirillales_Incertae_Sedis	Stella	Rare
ASV_53853	14	Connector	Acidobacteriota	Holophagae	Subgroup_7	Subgroup_7	Subgroup_7	Rare
ASV_53841	14	Connector	Proteobacteria	Gammaproteobacteria	Gammaproteobacteria_I	Unknown_Family	Acidibacter	Rare

					ncertae_Sedis			
ASV_53584	N	Connector	Bacteroidota	Bacteroidia	Sphingobacteriales	KD3-93	KD3-93	Rare
ASV_53425	21	Connector	Proteobacteria	Gammaproteobacteria	Xanthomonadales	Rhodanobacteraceae	Rhodanobacter	Rare
ASV_52358	12	Connector	Proteobacteria	Alphaproteobacteria	Tistrellales	Geminicoccaceae	Geminicoccus	Rare
ASV_50778	14	Connector	Acidobacteriota	Acidobacteriae	Bryobacterales	Bryobacteraceae	Bryobacter	Rare
ASV_50257	N	Connector	Actinobacteriota	Actinobacteria	Propionibacteriales	Nocardioidaceae	Nocardioides	Rare
ASV_50216	15	Connector	Actinobacteriota	Thermoleophilia	Solirubrobacterales	67-14	67-14	Rare
ASV_50068	14	Connector	Proteobacteria	Alphaproteobacteria	Rhizobiales	Xanthobacteraceae	unclassified	Rare
ASV_4984	14	Connector	Actinobacteriota	Acidimicrobiia	Microtrichales	Iamiaceae	Iamia	Rare
ASV_48076	14	Connector	Chloroflexi	Chloroflexia	Kallotenuales	AKIW781	AKIW781	Rare
ASV_47667	21	Connector	Proteobacteria	Gammaproteobacteria	Burkholderiales	Oxalobacteraceae	Noviherbaspirillum	Rare
ASV_46987	14	Connector	Chloroflexi	KD4-96	KD4-96	KD4-96	KD4-96	Rare
ASV_45496	15	Connector	Proteobacteria	Gammaproteobacteria	Burkholderiales	TRA3-20	TRA3-20	Rare
ASV_45469	15	Connector	Proteobacteria	Gammaproteobacteria	Xanthomonadales	Xanthomonadaceae	Arenimonas	Rare
ASV_45462	N	Connector	Acidobacteriota	Vicinamibacteria	Vicinamibacterales	uncultured	uncultured	Rare
ASV_45308	21	Connector	Actinobacteriota	Actinobacteria	Propionibacteriales	Nocardioidaceae	Nocardioides	Rare
ASV_44205	12	Connector	Gemmatimonadota	Gemmatimonadetes	Gemmatimonadales	Gemmatimonadaceae	Roseisolibacter	Rare
ASV_4346	N	Connector	Chloroflexi	Anaerolineae	SBR1031	SBR1031	SBR1031	Rare
ASV_42892	N	Connector	Actinobacteriota	Actinobacteria	Micromonosporales	Micromonosporaceae	Micromonospora	Rare
ASV_4285	14	Connector	Acidobacteriota	Vicinamibacteria	Vicinamibacterales	uncultured	uncultured	Rare
ASV_4145	N	Connector	Acidobacteriota	Acidobacteriae	Solibacterales	Solibacteraceae	Candidatus_Solibacter	Rare
ASV_41432	21	Connector	Chloroflexi	TK10	TK10	TK10	TK10	Rare
ASV_41269	12	Connector	Actinobacteriota	Thermoleophilia	Solirubrobacterales	67-14	67-14	Rare
ASV_41133	N	Connector	Myxococcota	Polyangia	Polyangiales	Polyangiaceae	Sorangium	Rare
ASV_41019	14	Connector	Chloroflexi	Chloroflexia	Thermomicrobiales	JG30-KF-CM45	JG30-KF-CM45	Rare
ASV_40672	15	Connector	Actinobacteriota	Actinobacteria	Propionibacteriales	Nocardioidaceae	Nocardioides	Rare
ASV_39022	15	Connector	Gemmatimonadota	Gemmatimonadetes	Gemmatimonadales	Gemmatimonadaceae	Gemmatimonas	Rare
ASV_38001	21	Connector	Actinobacteriota	Actinobacteria	Propionibacteriales	Nocardioidaceae	Nocardioides	Rare
ASV_37476	14	Connector	Proteobacteria	Gammaproteobacteria	Burkholderiales	Burkholderiaceae	Limnobacter	Rare

ASV_36013	15	Connector	Proteobacteria	Alphaproteobacteria	Rhizobiales	Rhizobiaceae	unclassified_Rhizobiaceae	Rare
ASV_35636	12	Connector	Myxococcota	Polyangia	Polyangiales	BIrii41	BIrii41	Rare
ASV_34618	21	Connector	Actinobacteriota	Actinobacteria	Micrococcales	Promicromonosporaceae	Cellulosimicrobium	Rare
ASV_3427	14	Connector	Proteobacteria	Gammaproteobacteria	Gammaproteobacteria_Incertae_Sedis	Unknown_Family	Acidibacter	Rare
ASV_33187	15	Connector	Proteobacteria	Gammaproteobacteria	Burkholderiales	SC-I-84	SC-I-84	Rare
ASV_32548	14	Connector	Chloroflexi	Chloroflexia	Thermomicrobiales	JG30-KF-CM45	JG30-KF-CM45	Rare
ASV_32125	15	Connector	Actinobacteriota	Actinobacteria	Frankiales	Nakamurellaceae	Nakamurella	Rare
ASV_31418	N	Connector	Proteobacteria	Gammaproteobacteria	Gammaproteobacteria_Incertae_Sedis	Unknown_Family	Acidibacter	Rare
ASV_30899	14	Connector	Proteobacteria	Alphaproteobacteria	uncultured	uncultured	uncultured	Rare
ASV_30438	12	Connector	Actinobacteriota	Thermoleophilia	Solirubrobacterales	Solirubrobacteraceae	Conexibacter	Rare
ASV_29489	11	Connector	Proteobacteria	Alphaproteobacteria	Caulobacterales	Caulobacteraceae	Brevundimonas	Rare
ASV_29156	N	Connector	Bacteroidota	Bacteroidia	Chitinophagales	Chitinophagaceae	Flavitalea	Rare
ASV_27673	14	Connector	Proteobacteria	Gammaproteobacteria	Xanthomonadales	Xanthomonadaceae	Luteimonas	Rare
ASV_2632	15	Connector	Acidobacteriota	Vicinamibacteria	Vicinamibacterales	Vicinamibacteraceae	Vicinamibacteraceae	Rare
ASV_25890	15	Connector	Proteobacteria	Gammaproteobacteria	Burkholderiales	TRA3-20	TRA3-20	Rare
ASV_25835	14	Connector	Proteobacteria	Gammaproteobacteria	Pseudomonadales	Moraxellaceae	Acinetobacter	Rare
ASV_25323	14	Connector	Actinobacteriota	Thermoleophilia	Solirubrobacterales	67-14	67-14	Rare
ASV_24076	12	Connector	Bacteroidota	Bacteroidia	Chitinophagales	Chitinophagaceae	Terrimonas	Rare
ASV_23618	N	Connector	Proteobacteria	Alphaproteobacteria	Caulobacterales	Caulobacteraceae	uncultured	Rare
ASV_23368	14	Connector	Acidobacteriota	Vicinamibacteria	Vicinamibacterales	uncultured	uncultured	Rare
ASV_22668	21	Connector	Chloroflexi	Anaerolineae	SBR1031	A4b	A4b	Rare
ASV_22528	12	Connector	Bacteroidota	Bacteroidia	Chitinophagales	Chitinophagaceae	uncultured	Rare
ASV_21742	21	Connector	Proteobacteria	Gammaproteobacteria	Burkholderiales	Burkholderiaceae	Lautropia	Rare
ASV_20831	15	Connector	Firmicutes	Bacilli	Alicyclobacillales	Alicyclobacillaceae	Tumebacillus	Rare
ASV_20680	N	Connector	Firmicutes	Clostridia	Peptostreptococcales-Tissierellales	Peptostreptococcaceae	Sporacetigenium	Rare
ASV_1795	14	Connector	Acidobacteriota	Acidobacteriae	Solibacterales	Solibacteraceae	Candidatus_Solibacter	Rare

ASV_17389	14	Connector	Chloroflexi	Ktedonobacteria	C0119	C0119	C0119	Rare
ASV_16693	15	Connector	Actinobacteriota	Actinobacteria	Micrococcales	Cellulomonadaceae	Actinotalea	Rare
ASV_14703	14	Connector	Actinobacteriota	Actinobacteria	Propionibacteriales	Nocardioidaceae	Nocardioides	Rare
ASV_1366	15	Connector	Acidobacteriota	Vicinamibacteria	Vicinamibacterales	Vicinamibacteraceae	Vicinamibacteraceae	Rare
ASV_12533	15	Connector	Cyanobacteria	Sericytochromatia	Sericytochromatia	Sericytochromatia	Sericytochromatia	Rare
ASV_11854	14	Connector	Actinobacteriota	Actinobacteria	unclassified	unclassified	unclassified	Rare
ASV_109746	14	Connector	Proteobacteria	Gammaproteobacteria	Burkholderiales	Comamonadaceae	Rhizobacter	Rare
ASV_108883	21	Connector	Gemmatimonadota	Gemmatimonadetes	Gemmatimonadales	Gemmatimonadaceae	Gemmatimonas	Rare
ASV_108642	N	Connector	Chloroflexi	Gitt-GS-136	Gitt-GS-136	Gitt-GS-136	Gitt-GS-136	Rare
ASV_108543	15	Connector	Proteobacteria	Alphaproteobacteria	Sphingomonadales	Sphingomonadaceae	Sphingomonas	Rare
ASV_107278	11	Connector	Proteobacteria	Gammaproteobacteria	Xanthomonadales	Xanthomonadaceae	Thermomonas	Rare
ASV_106559	21	Connector	Actinobacteriota	Actinobacteria	Micrococcales	Cellulomonadaceae	unclassified	Rare
ASV_106254	14	Connector	Myxococcota	Polyangia	Haliangiales	Haliangiaceae	Haliangium	Rare
ASV_10615	14	Connector	Planctomycetota	Planctomycetes	Isosphaerales	Isosphaeraceae	uncultured	Rare
ASV_104963	12	Connector	Armatimonadota	uncultured	uncultured	uncultured	uncultured	Rare
ASV_104937	14	Connector	Gemmatimonadota	S0134_terrestrial_group	S0134_terrestrial_group	S0134_terrestrial_group	S0134_terrestrial_group	Rare
ASV_104890	N	Connector	Planctomycetota	Phycisphaerae	Phycisphaerales	Phycisphaeraceae	SM1A02	Rare
ASV_10486	15	Connector	Proteobacteria	Gammaproteobacteria	Xanthomonadales	Xanthomonadaceae	Lysobacter	Rare
ASV_104587	21	Connector	Proteobacteria	Gammaproteobacteria	Ectothiorhodospirales	Thioalkalispiraceae	uncultured	Rare
ASV_104538	14	Connector	Planctomycetota	Phycisphaerae	Phycisphaerales	Phycisphaeraceae	SM1A02	Rare
ASV_103495	14	Connector	Myxococcota	Polyangia	Polyangiales	Polyangiaceae	Pajaroellobacter	Rare
ASV_102498	N	Connector	Proteobacteria	Alphaproteobacteria	Sphingomonadales	Sphingomonadaceae	Sphingomonas	Rare
ASV_102048	N	Connector	Proteobacteria	Gammaproteobacteria	Burkholderiales	Oxalobacteraceae	Massilia	Rare

Table S7. Taxa identified in the In treatment using Zi-Pi screening.

ASV	modu	Zi-Pi	Phylum	Class	Order	Family	Genus	Type
ASV_98501	N	Connector	Chloroflexi	Ktedonobacteria	C0119	C0119	C0119	Abundant
ASV_97730	10	Connector	unclassified	unclassified_Bacteria	unclassified_Bacteria	unclassified_Bacteria	unclassified_Bacteria	Abundant
ASV_97609	31	Connector	Actinobacteriota	Actinobacteria	Propionibacteriales	Nocardioidaceae	Nocardioides	Abundant
ASV_97181	4	Connector	Proteobacteria	Alphaproteobacteria	Rhizobiales	Xanthobacteraceae	unclassified_Xanthobacteraceae	Abundant
ASV_9703	11	Connector	Actinobacteriota	Actinobacteria	Propionibacteriales	Nocardioidaceae	Nocardioides	Abundant
ASV_9659	31	Connector	Actinobacteriota	Actinobacteria	Micrococcales	Micrococcaceae	unclassified_Micrococcaceae	Abundant
ASV_96022	10	Connector	Actinobacteriota	Actinobacteria	Frankiales	unclassified_Frankiales	unclassified_Frankiales	Abundant
ASV_95607	N	Connector	Gemmatimonadota	Gemmatimonadetes	Gemmatimonadales	Gemmatimonadaceae	uncultured	Abundant
ASV_95072	10	Module hub	Actinobacteriota	Actinobacteria	Micrococcales	Dermacoccaceae	Flexivirga	Abundant
ASV_94855	N	Connector	Actinobacteriota	Thermoleophilia	Gaiellales	uncultured	uncultured	Abundant
ASV_90175	10	Module hub	Actinobacteriota	Actinobacteria	Micrococcales	Micrococcaceae	unclassified_Micrococcaceae	Abundant
ASV_87974	4	Connector	Chloroflexi	Chloroflexia	Chloroflexales	Chloroflexaceae	FFCH7168	Abundant
ASV_87091	11	Connector	Acidobacteriota	Thermoanaerobaculia	Thermoanaerobaculales	Thermoanaerobaculaceae	Subgroup_10	Abundant
ASV_85892	N	Connector	Myxococcota	Polyangia	Polyangiales	Polyangiaceae	Pajaroellobacter	Abundant
ASV_85405	10	Module hub	Chloroflexi	Anaerolineae	SBR1031	A4b	A4b	Abundant
ASV_85333	31	Connector	Bacteroidota	Bacteroidia	Chitinophagales	Chitinophagaceae	Puia	Abundant
ASV_83083	11	Connector	Gemmatimonadota	Gemmatimonadetes	Gemmatimonadales	Gemmatimonadaceae	Gemmatimonas	Abundant
ASV_82598	11	Connector	Bacteroidota	Bacteroidia	Chitinophagales	Chitinophagaceae	Flavisolibacter	Abundant
ASV_8230	31	Connector	Chloroflexi	Ktedonobacteria	C0119	C0119	C0119	Abundant
ASV_81727	N	Connector	Acidobacteriota	Vicinamibacteria	Vicinamibacterales	uncultured	uncultured	Abundant
ASV_81227	N	Connector	Gemmatimonadota	Gemmatimonadetes	Gemmatimonadales	Gemmatimonadaceae	Gemmatimonas	Abundant
ASV_80632	10	Module hub	Actinobacteriota	Actinobacteria	Micrococcales	Microbacteriaceae	unclassified_Microbacteriaceae	Abundant
ASV_80424	31	Connector	Proteobacteria	Gammaproteobacteria	Burkholderiales	SC-I-84	SC-I-84	Abundant
ASV_79759	31	Connector	Actinobacteriota	Actinobacteria	Micrococcales	Cellulomonadaceae	Actinotalea	Abundant
ASV_79719	11	Connector	Proteobacteria	Alphaproteobacteria	Sphingomonadales	Sphingomonadaceae	unclassified_Sphingomonadaceae	Abundant
ASV_79264	10	Connector	Proteobacteria	Alphaproteobacteria	Rhizobiales	D05-2	D05-2	Abundant

ASV_7854	31	Connector	Actinobacteriota	Actinobacteria	Micrococcales	Microbacteriaceae	unclassified_Microbacteriaceae	Abundant
ASV_78498	31	Module hub	Actinobacteriota	Thermoleophilia	Solirubrobacterales	67-14	67-14	Abundant
ASV_77713	N	Connector	Proteobacteria	Alphaproteobacteria	Rhizobiales	Kaistiaceae	Kaistia	Abundant
ASV_76556	11	Connector	Acidobacteriota	Vicinamibacteria	Vicinamibacterales	Vicinamibacteraceae	Vicinamibacteraceae	Abundant
ASV_75855	10	Module hub	Actinobacteriota	Actinobacteria	Micrococcales	Micrococcaceae	Arthrobacter	Abundant
ASV_75434	N	Connector	Myxococcota	Myxococcia	Myxococcales	Myxococcaceae	P3OB-42	Abundant
ASV_72717	10	Connector	Chloroflexi	OLB14	OLB14	OLB14	OLB14	Abundant
ASV_72105	N	Connector	Actinobacteriota	Acidimicrobiia	Microtrichales	uncultured	uncultured	Abundant
ASV_71980	11	Connector	Chloroflexi	Anaerolineae	SBR1031	A4b	A4b	Abundant
ASV_71251	11	Connector	Chloroflexi	Chloroflexia	Chloroflexales	Roseiflexaceae	uncultured	Abundant
ASV_71001	31	Connector	Proteobacteria	Gammaproteobacteria	Pseudomonadales	Pseudomonadaceae	Pseudomonas	Abundant
ASV_69373	11	Connector	Actinobacteriota	Thermoleophilia	Gaiellales	uncultured	uncultured	Abundant
ASV_68313	27	Connector	Actinobacteriota	Thermoleophilia	Gaiellales	uncultured	uncultured	Abundant
ASV_67947	11	Connector	Myxococcota	Polyangia	Haliangiales	Haliangiaceae	Haliangium	Abundant
ASV_67865	11	Connector	Chloroflexi	Anaerolineae	SBR1031	A4b	A4b	Abundant
ASV_67703	10	Connector	Acidobacteriota	Vicinamibacteria	Vicinamibacterales	Vicinamibacteraceae	Vicinamibacteraceae	Abundant
ASV_67510	10	Connector	Proteobacteria	Alphaproteobacteria	Rhizobiales	Rhizobiales_Incertae_Sedis	Nordella	Abundant
ASV_664	10	Connector	Actinobacteriota	Thermoleophilia	Gaiellales	uncultured	uncultured	Abundant
ASV_6588	10	Module hub	Actinobacteriota	Actinobacteria	Micrococcales	Micrococcaceae	unclassified_Micrococcaceae	Abundant
ASV_65868	4	Connector	Actinobacteriota	Actinobacteria	Micrococcales	Intrasporangiaceae	unclassified_Intrasporangiaceae	Abundant
ASV_657	10	Connector	Actinobacteriota	Actinobacteria	Streptosporangiales	Thermomonosporaceae	Actinomadura	Abundant
ASV_65174	10	Connector	Proteobacteria	Alphaproteobacteria	Rhizobiales	Rhizobiaceae	Mesorhizobium	Abundant
ASV_64067	N	Connector	Proteobacteria	Gammaproteobacteria	Xanthomonadales	Xanthomonadaceae	Arenimonas	Abundant
ASV_63754	31	Connector	Actinobacteriota	Thermoleophilia	Solirubrobacterales	67-14	67-14	Abundant
ASV_6363	N	Connector	Proteobacteria	Alphaproteobacteria	Dongiales	Dongiaceae	Dongia	Abundant
ASV_62650	11	Connector	Actinobacteriota	Thermoleophilia	Solirubrobacterales	Solirubrobacteraceae	Solirubrobacter	Abundant
ASV_61885	4	Connector	Proteobacteria	Gammaproteobacteria	Burkholderiales	SC-I-84	SC-I-84	Abundant
ASV_61487	11	Connector	Actinobacteriota	Actinobacteria	Frankiales	Geodermatophilaceae	Blastococcus	Abundant
ASV_6110	10	Connector	Actinobacteriota	Thermoleophilia	Solirubrobacterales	Solirubrobacteraceae	JCM_18997	Abundant

ASV_57264	N	Connector	Proteobacteria	Gammaproteobacteria	Burkholderiales	A21b	A21b	Abundant
ASV_57024	11	Connector	Chloroflexi	Chloroflexia	Thermomicrobiales	AKYG1722	AKYG1722	Abundant
ASV_56543	N	Connector	Patescibacteria	Microgenomatia	Candidatus_Roizmanbacteria	Candidatus_Roizmanbacteria	Candidatus_Roizmanbacteria	Abundant
ASV_56433	11	Module hub	Actinobacteriota	Acidimicrobiia	IMCC26256	IMCC26256	IMCC26256	Abundant
ASV_55612	10	Connector	Gemmatimonadota	Gemmatimonadetes	Gemmatimonadales	Gemmatimonadaceae	Gemmatimonas	Abundant
ASV_54803	N	Connector	Chloroflexi	Chloroflexia	Chloroflexales	Roseiflexaceae	uncultured	Abundant
ASV_53383	27	Connector	Proteobacteria	Gammaproteobacteria	Burkholderiales	SC-I-84	SC-I-84	Abundant
ASV_53309	31	Module hub	Actinobacteriota	Actinobacteria	Micrococcales	Microbacteriaceae	unclassified_Microbacteriaceae	Abundant
ASV_52483	17	Connector	Proteobacteria	Gammaproteobacteria	Xanthomonadales	Rhodanobacteraceae	Dokdonella	Abundant
ASV_52146	11	Connector	Actinobacteriota	Acidimicrobiia	IMCC26256	IMCC26256	IMCC26256	Abundant
ASV_51885	11	Connector	Chloroflexi	Ktedonobacteria	Ktedonobacterales	JG30-KF-AS9	JG30-KF-AS9	Abundant
ASV_51747	10	Module hub	Chloroflexi	Anaerolineae	Anaerolineales	Anaerolineaceae	uncultured	Abundant
ASV_50892	N	Connector	Actinobacteriota	Actinobacteria	Micromonosporales	Micromonosporaceae	unclassified	Abundant
ASV_49812	10	Connector	Proteobacteria	Gammaproteobacteria	Xanthomonadales	Rhodanobacteraceae	Rhodanobacter	Abundant
ASV_49648	11	Connector	Gemmatimonadota	Gemmatimonadetes	Gemmatimonadales	Gemmatimonadaceae	uncultured	Abundant
ASV_49265	11	Connector	Actinobacteriota	Actinobacteria	Micromonosporales	Micromonosporaceae	Actinoplanes	Abundant
ASV_48765	N	Connector	Proteobacteria	Alphaproteobacteria	Rhizobiales	Xanthobacteraceae	Bradyrhizobium	Abundant
ASV_48598	N	Connector	Verrucomicrobiota	Chlamydiae	Chlamydiales	Parachlamydiaceae	Neochlamydia	Abundant
ASV_47766	27	Connector	Chloroflexi	TK10	TK10	TK10	TK10	Abundant
ASV_47583	10	Connector	Actinobacteriota	Actinobacteria	Propionibacteriales	Nocardioidaceae	Nocardioides	Abundant
ASV_46807	17	Connector	Myxococcota	Myxococcia	Myxococcales	Myxococcaceae	Archangium	Abundant
ASV_46801	31	Connector	Actinobacteriota	Actinobacteria	Corynebacteriales	Mycobacteriaceae	Mycobacterium	Abundant
ASV_46683	10	Connector	Acidobacteriota	Blastocatellia	Blastocatellales	Blastocatellaceae	unclassified	Abundant
ASV_4657	11	Connector	Actinobacteriota	Actinobacteria	Corynebacteriales	Mycobacteriaceae	Mycobacterium	Abundant
ASV_44550	11	Connector	Actinobacteriota	Actinobacteria	Micrococcales	Microbacteriaceae	Cryobacterium	Abundant
ASV_44219	10	Connector	Actinobacteriota	Actinobacteria	Frankiales	Geodermatophilaceae	Geodermatophilus	Abundant
ASV_44046	N	Connector	Chloroflexi	Anaerolineae	SBR1031	A4b	A4b	Abundant
ASV_44014	N	Connector	Actinobacteriota	Actinobacteria	Propionibacteriales	Nocardioidaceae	Nocardioides	Abundant

ASV_43260	11	Connector	Actinobacteriota	Thermoleophilia	Gaiellales	uncultured	uncultured	Abundant
ASV_43019	N	Connector	Firmicutes	Bacilli	Bacillales	Planococcaceae	Sporosarcina	Abundant
ASV_41722	N	Connector	Chloroflexi	Ktedonobacteria	C0119	C0119	C0119	Abundant
ASV_40508	N	Connector	Actinobacteriota	Actinobacteria	Micromonosporales	Micromonosporaceae	Micromonospora	Abundant
ASV_40503	31	Connector	Chloroflexi	Ktedonobacteria	B10-SB3A	B10-SB3A	B10-SB3A	Abundant
ASV_40160	10	Module hub	Actinobacteriota	Actinobacteria	Micrococcales	Intrasporangiaceae	unclassified	Abundant
ASV_39847	10	Connector	Gemmatimonadota	Gemmatimonadetes	Gemmatimonadales	Gemmatimonadaceae	uncultured	Abundant
ASV_39106	N	Connector	Actinobacteriota	Actinobacteria	Propionibacteriales	Nocardioidaceae	Nocardioides	Abundant
ASV_38764	10	Connector	Proteobacteria	Alphaproteobacteria	Rhizobiales	Xanthobacteraceae	Pseudolabrys	Abundant
ASV_37485	11	Connector	Chloroflexi	KD4-96	KD4-96	KD4-96	KD4-96	Abundant
ASV_36988	4	Connector	Actinobacteriota	Acidimicrobiia	IMCC26256	IMCC26256	IMCC26256	Abundant
ASV_36386	11	Connector	Acidobacteriota	Acidobacteriae	Bryobacterales	Bryobacteraceae	Bryobacter	Abundant
ASV_36286	10	Connector	Acidobacteriota	Vicinamibacteria	Vicinamibacterales	uncultured	uncultured	Abundant
ASV_34682	4	Connector	Acidobacteriota	Blastocatellia	Blastocatellales	Blastocatellaceae	JGI_0001001-H03	Abundant
ASV_30969	10	Connector	Acidobacteriota	Vicinamibacteria	Vicinamibacterales	Vicinamibacteraceae	Vicinamibacteraceae	Abundant
ASV_30246	10	Connector	Proteobacteria	Gammaproteobacteria	Burkholderiales	SC-I-84	SC-I-84	Abundant
ASV_29994	N	Connector	Chloroflexi	Anaerolineae	SBR1031	A4b	A4b	Abundant
ASV_29817	17	Connector	Proteobacteria	Alphaproteobacteria	Acetobacterales	Acetobacteraceae	uncultured	Abundant
ASV_28313	10	Connector	Cyanobacteria	Cyanobacteriia	Chloroplast	Chloroplast	Chloroplast	Abundant
ASV_26144	31	Connector	Proteobacteria	Alphaproteobacteria	Sphingomonadales	Sphingomonadaceae	Sphingomonas	Abundant
ASV_23241	31	Connector	Proteobacteria	Gammaproteobacteria	Burkholderiales	Nitrosomonadaceae	Ellin6067	Abundant
ASV_2248	31	Connector	Actinobacteriota	Acidimicrobiia	Microtrichales	uncultured	uncultured	Abundant
ASV_21851	27	Connector	Actinobacteriota	Actinobacteria	Micrococcales	Intrasporangiaceae	unclassified	Abundant
ASV_21711	11	Module hub	Actinobacteriota	Actinobacteria	Micrococcales	Intrasporangiaceae	unclassified	Abundant
ASV_21644	10	Module hub	Acidobacteriota	Acidobacteriae	Bryobacterales	Bryobacteraceae	Bryobacter	Abundant
ASV_21106	11	Connector	Gemmatimonadota	Gemmatimonadetes	Gemmatimonadales	Gemmatimonadaceae	Gemmatimonas	Abundant
ASV_20752	11	Connector	Chloroflexi	KD4-96	KD4-96	KD4-96	KD4-96	Abundant
ASV_19698	N	Connector	Actinobacteriota	Thermoleophilia	Solirubrobacterales	Solirubrobacteraceae	Conexibacter	Abundant
ASV_18756	27	Connector	Actinobacteriota	Thermoleophilia	Gaiellales	uncultured	uncultured	Abundant

ASV_17633	11	Connector	Proteobacteria	Alphaproteobacteria	Defluviicoccales	Defluviicoccaceae	Defluviicoccus	Abundant
ASV_17335	10	Connector	Proteobacteria	Gammaproteobacteria	Xanthomonadales	Xanthomonadaceae	Arenimonas	Abundant
ASV_16873	10	Connector	Chloroflexi	KD4-96	KD4-96	KD4-96	KD4-96	Abundant
ASV_16508	4	Connector	Chloroflexi	Chloroflexia	Thermomicrobiales	JG30-KF-CM45	JG30-KF-CM45	Abundant
ASV_15216	11	Connector	Chloroflexi	KD4-96	KD4-96	KD4-96	KD4-96	Abundant
ASV_14076	N	Connector	Actinobacteriota	Acidimicrobiia	IMCC26256	IMCC26256	IMCC26256	Abundant
ASV_12680	N	Connector	Chloroflexi	Chloroflexia	Thermomicrobiales	JG30-KF-CM45	JG30-KF-CM45	Abundant
ASV_11641	31	Connector	Proteobacteria	Alphaproteobacteria	Sphingomonadales	Sphingomonadaceae	unclassified	Abundant
ASV_11554	10	Connector	Proteobacteria	Alphaproteobacteria	Sphingomonadales	Sphingomonadaceae	Ellin6055	Abundant
ASV_11024	N	Connector	Proteobacteria	Gammaproteobacteria	Burkholderiales	Nitrosomonadaceae	MND1	Abundant
ASV_110054	11	Connector	Chloroflexi	Ktedonobacteria	Ktedonobacterales	JG30-KF-AS9	JG30-KF-AS9	Abundant
ASV_107535	N	Connector	Planctomycetota	Planctomycetes	Isosphaerales	Isosphaeraceae	Isosphaera	Abundant
ASV_107134	11	Connector	Actinobacteriota	Thermoleophilia	Gaiellales	uncultured	uncultured	Abundant
ASV_106932	10	Module hub	Proteobacteria	Gammaproteobacteria	Burkholderiales	SC-I-84	SC-I-84	Abundant
ASV_104137	27	Connector	Actinobacteriota	Actinobacteria	Propionibacteriales	Nocardioidaceae	Marmoricola	Abundant
ASV_103258	11	Connector	Proteobacteria	Alphaproteobacteria	Reyranellales	Reyranellaceae	uncultured	Abundant
ASV_10322	N	Connector	Acidobacteriota	Vicinamibacteria	Vicinamibacterales	uncultured	uncultured	Abundant
ASV_102609	N	Connector	Myxococcota	Polyangia	Haliangiales	Haliangiaceae	Haliangium	Abundant
ASV_102477	11	Connector	Proteobacteria	Alphaproteobacteria	Rhizobiales	Amb-16S-1323	Amb-16S-1323	Abundant
ASV_10231	11	Connector	Gemmatimonadota	Gemmatimonadetes	Gemmatimonadales	Gemmatimonadaceae	Gemmatimonas	Abundant
ASV_10208	11	Connector	Proteobacteria	Gammaproteobacteria	Steroidobacterales	Steroidobacteraceae	Steroidobacter	Abundant
ASV_100724	27	Connector	Proteobacteria	Gammaproteobacteria	Xanthomonadales	Rhodanobacteraceae	Rhodanobacter	Abundant
ASV_100336	N	Connector	Actinobacteriota	Thermoleophilia	Solirubrobacterales	67-14	67-14	Abundant
ASV_100268	11	Connector	Actinobacteriota	Acidimicrobiia	Microtrichales	Ilumatobacteraceae	Ilumatobacter	Abundant
ASV_99677	10	Connector	Proteobacteria	Gammaproteobacteria	Burkholderiales	SC-I-84	SC-I-84	Rare
ASV_99591	N	Connector	Proteobacteria	Gammaproteobacteria	Steroidobacterales	Steroidobacteraceae	Steroidobacter	Rare
ASV_98634	N	Connector	Verrucomicrobiota	Verrucomicrobiae	Opitutales	Opitutaceae	Opitutus	Rare
ASV_98430	10	Connector	Firmicutes	Bacilli	Alicyclobacillales	Alicyclobacillaceae	Tumebacillus	Rare
ASV_97881	11	Connector	Chloroflexi	JG30-KF-CM66	JG30-KF-CM66	JG30-KF-CM66	JG30-KF-CM66	Rare

ASV_97709	N	Connector	Bacteroidota	Bacteroidia	Chitinophagales	Chitinophagaceae	uncultured	Rare
ASV_96891	4	Connector	Actinobacteriota	Actinobacteria	Corynebacteriales	Mycobacteriaceae	Mycobacterium	Rare
ASV_96535	10	Connector	Chloroflexi	Chloroflexia	Thermomicrobiales	Thermomicrobiaceae	Nitrolancea	Rare
ASV_95713	31	Connector	Armatimonadota	Chthonomonadetes	Chthonomonadales	Chthonomonadaceae	Chthonomonas	Rare
ASV_95670	N	Connector	unclassified	unclassified_Bacteria	unclassified_Bacteria	unclassified_Bacteria	unclassified_Bacteria	Rare
ASV_95156	N	Connector	Gemmatimonadota	Gemmatimonadetes	Gemmatimonadales	Gemmatimonadaceae	uncultured	Rare
ASV_95115	10	Connector	Myxococcota	Polyangia	Polyangiales	Polyangiaceae	Pajaroellobacter	Rare
ASV_94987	4	Connector	Bacteroidota	Bacteroidia	Chitinophagales	Chitinophagaceae	uncultured	Rare
ASV_94923	N	Connector	Acidobacteriota	Vicinamibacteria	Vicinamibacterales	uncultured	uncultured	Rare
ASV_94760	11	Module hub	Actinobacteriota	Actinobacteria	Propionibacteriales	Nocardioidaceae	Nocardioides	Rare
ASV_94124	4	Connector	Fibrobacterota	Fibrobacteria	Fibrobacterales	Fibrobacteraceae	possible_genus_04	Rare
ASV_93825	N	Connector	Bacteroidota	Bacteroidia	Flavobacteriales	Flavobacteriaceae	Flavobacterium	Rare
ASV_93361	10	Connector	Proteobacteria	Gammaproteobacteria	Burkholderiales	SC-I-84	SC-I-84	Rare
ASV_91823	4	Connector	Actinobacteriota	Acidimicrobiia	IMCC26256	IMCC26256	IMCC26256	Rare
ASV_91531	31	Connector	Gemmatimonadota	Gemmatimonadetes	Gemmatimonadales	Gemmatimonadaceae	uncultured	Rare
ASV_91205	N	Connector	Acidobacteriota	Vicinamibacteria	Vicinamibacterales	uncultured	uncultured	Rare
ASV_90590	31	Connector	Actinobacteriota	Actinobacteria	Corynebacteriales	Mycobacteriaceae	Mycobacterium	Rare
ASV_90438	17	Connector	Chloroflexi	Chloroflexia	Kallotenuales	AKIW781	AKIW781	Rare
ASV_90130	2	Connector	Proteobacteria	Alphaproteobacteria	unclassified	unclassified	unclassified	Rare
ASV_89774	N	Connector	Proteobacteria	Gammaproteobacteria	Diplorickettsiales	Diplorickettsiaceae	uncultured	Rare
ASV_89071	17	Connector	Myxococcota	Polyangia	Polyangiales	Sandaracinaceae	uncultured	Rare
ASV_88729	4	Connector	Proteobacteria	Gammaproteobacteria	Pseudomonadales	Pseudomonadaceae	Pseudomonas	Rare
ASV_88459	10	Connector	Acidobacteriota	Acidobacteriae	Bryobacterales	Bryobacteraceae	Bryobacter	Rare
ASV_8838	11	Connector	Proteobacteria	Alphaproteobacteria	Rhizobiales	Hyphomicrobiaceae	Pedomicrobium	Rare
ASV_8780	N	Connector	Proteobacteria	Gammaproteobacteria	Pseudomonadales	Pseudomonadaceae	Pseudomonas	Rare
ASV_87512	11	Connector	Actinobacteriota	Thermoleophilia	Gaiellales	uncultured	uncultured	Rare
ASV_87267	11	Module hub	Proteobacteria	Alphaproteobacteria	Caulobacterales	Caulobacteraceae	Phenylobacterium	Rare
ASV_86704	N	Connector	Gemmatimonadota	Longimicrobia	Longimicrobiales	Longimicrobiaceae	Longimicrobiaceae	Rare
ASV_86559	N	Connector	Chloroflexi	Ktedonobacteria	Ktedonobacterales	JG30-KF-AS9	JG30-KF-AS9	Rare

ASV_86452	N	Connector	Bacteroidota	Kapabacteria	Kapabacteriales	Kapabacteriales	Kapabacteriales	Rare
ASV_85755	N	Connector	Proteobacteria	Alphaproteobacteria	Acetobacterales	Acetobacteraceae	uncultured	Rare
ASV_85546	N	Connector	Actinobacteriota	Thermoleophilia	Gaiellales	uncultured	uncultured	Rare
ASV_84335	17	Connector	Actinobacteriota	Actinobacteria	Corynebacteriales	Mycobacteriaceae	Mycobacterium	Rare
ASV_84161	N	Connector	Chloroflexi	OLB14	OLB14	OLB14	OLB14	Rare
ASV_83758	N	Connector	Proteobacteria	Gammaproteobacteria	Xanthomonadales	Xanthomonadaceae	Arenimonas	Rare
ASV_83542	10	Connector	Actinobacteriota	Actinobacteria	Micrococcales	Microbacteriaceae	unclassified	Rare
ASV_83244	17	Connector	Gemmatimonadota	Gemmatimonadetes	Gemmatimonadales	Gemmatimonadaceae	Gemmatimonas	Rare
ASV_83241	N	Connector	Chloroflexi	Chloroflexia	Thermomicrobiales	JG30-KF-CM45	JG30-KF-CM45	Rare
ASV_82112	N	Connector	Actinobacteriota	Thermoleophilia	Solirubrobacterales	Solirubrobacteraceae	JCM_18997	Rare
ASV_81656	10	Connector	Actinobacteriota	Acidimicrobiia	Microtrichales	Ilumatobacteraceae	CL500-29_marine_group	Rare
ASV_8161	27	Connector	Proteobacteria	Gammaproteobacteria	Burkholderiales	Nitrosomonadaceae	Ellin6067	Rare
ASV_81597	N	Connector	Actinobacteriota	Acidimicrobiia	IMCC26256	IMCC26256	IMCC26256	Rare
ASV_81380	31	Connector	Verrucomicrobiota	Chlamydiae	Chlamydiales	Simkaniaceae	uncultured	Rare
ASV_81071	N	Connector	Proteobacteria	Gammaproteobacteria	Burkholderiales	Comamonadaceae	unclassified	Rare
ASV_81025	N	Connector	Firmicutes	Clostridia	Peptostreptococcales-Tissierellales	Peptostreptococcales-Tissierellales	Alkaliphilus	Rare
ASV_79920	11	Connector	Bacteroidota	Bacteroidia	Chitinophagales	Chitinophagaceae	uncultured	Rare
ASV_79524	11	Connector	Actinobacteriota	Acidimicrobiia	Microtrichales	Ilumatobacteraceae	uncultured	Rare
ASV_78480	17	Connector	Proteobacteria	Alphaproteobacteria	Rhizobiales	Xanthobacteraceae	Rhodoplanes	Rare
ASV_7686	N	Connector	Acidobacteriota	Acidobacteriae	Bryobacterales	Bryobacteraceae	Bryobacter	Rare
ASV_76739	10	Connector	Proteobacteria	Alphaproteobacteria	Rhizobiales	Xanthobacteraceae	uncultured	Rare
ASV_76689	10	Connector	Chloroflexi	Ktedonobacteria	Ktedonobacterales	JG30-KF-AS9	JG30-KF-AS9	Rare
ASV_76483	31	Connector	Actinobacteriota	Thermoleophilia	Solirubrobacterales	Solirubrobacteraceae	Solirubrobacter	Rare
ASV_7646	N	Connector	Myxococcota	Polyangia	Nannocystales	Nannocystaceae	Nannocystis	Rare
ASV_76107	11	Connector	Actinobacteriota	Actinobacteria	Frankiales	Nakamurellaceae	Nakamurella	Rare
ASV_75698	31	Connector	Actinobacteriota	Thermoleophilia	Solirubrobacterales	Solirubrobacteraceae	Conexibacter	Rare
ASV_75684	10	Connector	Proteobacteria	Gammaproteobacteria	Xanthomonadales	Xanthomonadaceae	Luteimonas	Rare
ASV_75093	N	Connector	Actinobacteriota	Acidimicrobiia	IMCC26256	IMCC26256	IMCC26256	Rare

ASV_7477	N	Connector	Actinobacteriota	Thermoleophilia	Gaiellales	uncultured	uncultured	Rare
ASV_74598	11	Connector	Chloroflexi	Ktedonobacteria	C0119	C0119	C0119	Rare
ASV_74497	11	Connector	Acidobacteriota	Acidobacteriae	Bryobacterales	Bryobacteraceae	Bryobacter	Rare
ASV_74245	31	Connector	Proteobacteria	Gammaproteobacteria	Burkholderiales	Oxalobacteraceae	unclassified	Rare
ASV_7400	10	Connector	unclassified	unclassified	unclassified	unclassified	unclassified	Rare
ASV_73252	N	Connector	Acidobacteriota	Vicinamibacteria	Vicinamibacterales	uncultured	uncultured	Rare
ASV_73109	N	Connector	Gemmatimonadota	Gemmatimonadetes	Gemmatimonadales	Gemmatimonadaceae	Gemmatimonas	Rare
ASV_73011	N	Connector	Actinobacteriota	Thermoleophilia	Solirubrobacterales	Solirubrobacteraceae	Conexibacter	Rare
ASV_72413	N	Connector	Actinobacteriota	Actinobacteria	Micrococcales	Microbacteriaceae	unclassified	Rare
ASV_72206	10	Connector	Proteobacteria	Gammaproteobacteria	Burkholderiales	SC-I-84	SC-I-84	Rare
ASV_72150	27	Connector	Proteobacteria	Alphaproteobacteria	Rhizobiales	Xanthobacteraceae	uncultured	Rare
ASV_71898	N	Connector	Chloroflexi	TK10	TK10	TK10	TK10	Rare
ASV_70754	N	Connector	Proteobacteria	Gammaproteobacteria	CCD24	CCD24	CCD24	Rare
ASV_70204	N	Connector	Proteobacteria	Gammaproteobacteria	Burkholderiales	SC-I-84	SC-I-84	Rare
ASV_69641	17	Connector	Proteobacteria	Gammaproteobacteria	Burkholderiales	Comamonadaceae	Leptothrix	Rare
ASV_69038	31	Connector	Actinobacteriota	Thermoleophilia	Gaiellales	uncultured	uncultured	Rare
ASV_68543	N	Connector	Proteobacteria	Gammaproteobacteria	Burkholderiales	SC-I-84	SC-I-84	Rare
ASV_6828	N	Connector	Proteobacteria	Gammaproteobacteria	Burkholderiales	Nitrosomonadaceae	Ellin6067	Rare
ASV_67886	N	Connector	Chloroflexi	TK10	TK10	TK10	TK10	Rare
ASV_67842	N	Connector	Actinobacteriota	Actinobacteria	Pseudonocardiales	Pseudonocardiaceae	Actinomycetospora	Rare
ASV_67355	4	Connector	Actinobacteriota	Thermoleophilia	Solirubrobacterales	67-14	67-14	Rare
ASV_67351	17	Connector	Firmicutes	Clostridia	Peptostreptococcales-Tissierellales	Peptostreptococcales-Tissierellales	Natronincola	Rare
ASV_66339	27	Connector	Firmicutes	Bacilli	Aneurinibacillales	Aneurinibacillaceae	Aneurinibacillus	Rare
ASV_66315	N	Connector	Actinobacteriota	Acidimicrobiia	Microtrichales	Iamiaceae	Iamia	Rare
ASV_66185	10	Connector	Proteobacteria	Alphaproteobacteria	Rhizobiales	Hyphomicrobiaceae	Pedomicrobium	Rare
ASV_65828	31	Connector	Patescibacteria	Kazania	Kazania	Kazania	Kazania	Rare
ASV_65176	4	Connector	Planctomycetota	Planctomycetes	Isosphaerales	Isosphaeraceae	uncultured	Rare
ASV_6503	4	Connector	Gemmatimonadota	BD2-	BD2-	BD2-11_terrestrial_group	BD2-11_terrestrial_group	Rare

				11_terrestrial_group	11_terrestrial_group			
ASV_64822	N	Connector	Actinobacteriota	Thermoleophilia	Solirubrobacterales	67-14	67-14	Rare
ASV_64310	11	Connector	Actinobacteriota	Actinobacteria	Corynebacteriales	Nocardiaceae	Smaragdicoccus	Rare
ASV_6393	N	Connector	Acidobacteriota	Vicinamibacteria	Vicinamibacterales	Vicinamibacteraceae	Vicinamibacteraceae	Rare
ASV_6372	2	Connector	Verrucomicrobiota	Verrucomicrobiae	Chthoniobacterales	Chthoniobacteraceae	Candidatus_Udaeobacter	Rare
ASV_63656	N	Connector	Gemmatimonadota	Gemmatimonadetes	Gemmatimonadales	Gemmatimonadaceae	uncultured	Rare
ASV_6354	17	Connector	Actinobacteriota	Actinobacteria	Corynebacteriales	Mycobacteriaceae	Mycobacterium	Rare
ASV_62964	N	Connector	Actinobacteriota	Actinobacteria	Micrococcales	Microbacteriaceae	unclassified	Rare
ASV_62909	N	Connector	Firmicutes	Clostridia	Peptostreptococcales-Tissierellales	Family_XI	unclassified	Rare
ASV_61589	N	Connector	Bacteroidota	Bacteroidia	Chitinophagales	Saprospiraceae	uncultured	Rare
ASV_61039	11	Connector	Chloroflexi	Anaerolineae	SBR1031	A4b	A4b	Rare
ASV_60301	11	Connector	Acidobacteriota	Vicinamibacteria	Vicinamibacterales	uncultured	uncultured	Rare
ASV_60034	11	Connector	Bacteroidota	Bacteroidia	Chitinophagales	Chitinophagaceae	Flavisolibacter	Rare
ASV_59931	N	Connector	Chloroflexi	Ktedonobacteria	C0119	C0119	C0119	Rare
ASV_59902	N	Connector	Chloroflexi	Ktedonobacteria	Ktedonobacterales	Ktedonobacteraceae	FCPS473	Rare
ASV_59700	10	Connector	Proteobacteria	Alphaproteobacteria	Rhizobiales	Xanthobacteraceae	Bradyrhizobium	Rare
ASV_59126	N	Connector	Firmicutes	Bacilli	Bacillales	Bacillaceae	Pseudogracilibacillus	Rare
ASV_5906	10	Connector	Proteobacteria	Gammaproteobacteria	Burkholderiales	TRA3-20	TRA3-20	Rare
ASV_5883	10	Connector	Chloroflexi	Anaerolineae	Caldilineales	Caldilineaceae	uncultured	Rare
ASV_58500	11	Connector	Chloroflexi	Ktedonobacteria	Ktedonobacterales	Ktedonobacteraceae	FCPS473	Rare
ASV_5847	N	Module hub	Proteobacteria	Alphaproteobacteria	Micropepsales	Micropepsaceae	uncultured	Rare
ASV_57010	11	Connector	Proteobacteria	Gammaproteobacteria	Xanthomonadales	Rhodanobacteraceae	Rhodanobacter	Rare
ASV_5624	N	Connector	Firmicutes	Bacilli	Bacillales	Bacillaceae	Bacillus	Rare
ASV_56187	11	Connector	Proteobacteria	Alphaproteobacteria	Sphingomonadales	Sphingomonadaceae	Sphingomonas	Rare
ASV_55567	4	Connector	Bacteroidota	Bacteroidia	Chitinophagales	Chitinophagaceae	Flavisolibacter	Rare
ASV_54387	27	Connector	Proteobacteria	Alphaproteobacteria	Rhizobiales	Xanthobacteraceae	unclassified	Rare
ASV_53119	4	Connector	Actinobacteriota	Actinobacteria	Micrococcales	Intrasporangiaceae	unclassified	Rare
ASV_5271	N	Connector	Acidobacteriota	Thermoanaerobaculia	Thermoanaerobaculales	Thermoanaerobaculaceae	Subgroup_10	Rare

ASV_52023	2	Connector	Firmicutes	Bacilli	Bacillales	Bacillaceae	uncultured	Rare
ASV_51883	11	Connector	Bacteroidota	Bacteroidia	Sphingobacteriales	AKYH767	AKYH767	Rare
ASV_51196	10	Connector	Firmicutes	Bacilli	Bacillales	Planococcaceae	Sporosarcina	Rare
ASV_50708	31	Connector	Acidobacteriota	Vicinamibacteria	Vicinamibacterales	uncultured	uncultured	Rare
ASV_50242	N	Connector	Patescibacteria	Saccharimonadia	Saccharimonadales	Saccharimonadaceae	TM7a	Rare
ASV_49523	11	Connector	Chloroflexi	Chloroflexia	Thermomicrobiales	JG30-KF-CM45	JG30-KF-CM45	Rare
ASV_49500	N	Module hub	Acidobacteriota	Acidobacteriae	Bryobacterales	Bryobacteraceae	Bryobacter	Rare
ASV_49197	N	Connector	Proteobacteria	Gammaproteobacteria	Burkholderiales	SC-I-84	SC-I-84	Rare
ASV_48076	N	Connector	Chloroflexi	Chloroflexia	Kallotenuales	AKIW781	AKIW781	Rare
ASV_47974	4	Connector	Acidobacteriota	Acidobacteriae	Acidobacteriales	Acidobacteriaceae_(Subgroup_1)	uncultured	Rare
ASV_46401	10	Connector	Abditibacteriota	Abditibacteria	Abditibacteriales	Abditibacteriaceae	Abditibacterium	Rare
ASV_46318	N	Connector	Chloroflexi	KD4-96	KD4-96	KD4-96	KD4-96	Rare
ASV_46202	4	Connector	Firmicutes	Clostridia	Lachnospirales	Lachnospiraceae	uncultured	Rare
ASV_4585	10	Connector	Proteobacteria	Gammaproteobacteria	Pseudomonadales	Pseudomonadaceae	Pseudomonas	Rare
ASV_44949	N	Connector	Myxococcota	Myxococcia	Myxococcales	Myxococcaceae	P3OB-42	Rare
ASV_44239	N	Connector	Proteobacteria	Gammaproteobacteria	Pseudomonadales	Moraxellaceae	Acinetobacter	Rare
ASV_44228	27	Connector	Acidobacteriota	Vicinamibacteria	Vicinamibacterales	uncultured	uncultured	Rare
ASV_44148	10	Connector	Proteobacteria	Alphaproteobacteria	Azospirillales	Inquilinaceae	Inquilinus	Rare
ASV_43921	N	Connector	Actinobacteriota	Actinobacteria	Corynebacteriales	Mycobacteriaceae	Mycobacterium	Rare
ASV_43485	10	Connector	Proteobacteria	Gammaproteobacteria	R7C24	R7C24	R7C24	Rare
ASV_42166	N	Connector	Proteobacteria	Gammaproteobacteria	Pseudomonadales	Pseudomonadaceae	Pseudomonas	Rare
ASV_41540	4	Connector	Proteobacteria	Gammaproteobacteria	Xanthomonadales	Xanthomonadaceae	Lysobacter	Rare
ASV_41331	11	Connector	Planctomycetota	BD7-11	BD7-11	BD7-11	BD7-11	Rare
ASV_40548	10	Connector	Firmicutes	Bacilli	Bacillales	Bacillaceae	Bacillus	Rare
ASV_40178	N	Connector	Proteobacteria	Gammaproteobacteria	Burkholderiales	Burkholderiaceae	Limnobacter	Rare
ASV_40154	27	Connector	Myxococcota	Polyangia	Polyangiales	BIrii41	BIrii41	Rare
ASV_39988	31	Connector	Proteobacteria	Alphaproteobacteria	Sphingomonadales	Sphingomonadaceae	unclassified	Rare
ASV_39678	10	Connector	Acidobacteriota	Vicinamibacteria	Vicinamibacterales	uncultured	uncultured	Rare
ASV_3934	N	Connector	Myxococcota	Polyangia	Polyangiales	Sandaracinaceae	uncultured	Rare

ASV_39268	N	Connector	Cyanobacteria	Vampirivibrionia	Vampirovibrionales	Vampirovibrionales	Vampirovibrionales	Rare
ASV_38797	4	Connector	Proteobacteria	Gammaproteobacteria	Burkholderiales	Comamonadaceae	unclassified_Comamonadaceae	Rare
ASV_38408	31	Module hub	Actinobacteriota	Actinobacteria	Micrococcales	Micrococcaceae	unclassified_Micrococcaceae	Rare
ASV_3814	N	Connector	Actinobacteriota	Thermoleophilia	Solirubrobacterales	67-14	67-14	Rare
ASV_37603	N	Connector	Gemmatimonadota	Gemmatimonadetes	Gemmatimonadales	Gemmatimonadaceae	uncultured	Rare
ASV_36867	31	Connector	Actinobacteriota	Actinobacteria	Micrococcales	Micrococcaceae	unclassified_Micrococcaceae	Rare
ASV_36820	N	Connector	Bacteroidota	Bacteroidia	Chitinophagales	Chitinophagaceae	uncultured	Rare
ASV_36699	27	Connector	Acidobacteriota	Vicinamibacteria	Vicinamibacterales	uncultured	uncultured	Rare
ASV_36384	N	Connector	Chloroflexi	Anaerolineae	SBR1031	A4b	A4b	Rare
ASV_35639	N	Connector	Actinobacteriota	Actinobacteria	Micromonosporales	Micromonosporaceae	Actinoplanes	Rare
ASV_35173	N	Connector	Chloroflexi	Ktedonobacteria	Ktedonobacterales	Ktedonobacteraceae	FCPS473	Rare
ASV_3411	N	Connector	Actinobacteriota	Thermoleophilia	Gaiellales	Gaiellaceae	Gaiella	Rare
ASV_33106	11	Connector	Proteobacteria	Gammaproteobacteria	Burkholderiales	Nitrosomonadaceae	MND1	Rare
ASV_32697	N	Connector	Firmicutes	Bacilli	Bacillales	Bacillaceae	unclassified_Bacillaceae	Rare
ASV_32278	31	Connector	Proteobacteria	Alphaproteobacteria	Caulobacterales	Caulobacteraceae	Brevundimonas	Rare
ASV_309	31	Connector	Bdellovibrionota	Bdellovibrionia	Bdellovibrionales	Bdellovibrionaceae	Bdellovibrio	Rare
ASV_30871	4	Connector	Patescibacteria	Saccharimonadia	Saccharimonadales	Saccharimonadales	Saccharimonadales	Rare
ASV_30639	11	Module hub	Actinobacteriota	Actinobacteria	Micrococcales	Micrococcaceae	unclassified_Micrococcaceae	Rare
ASV_30534	11	Connector	Proteobacteria	Gammaproteobacteria	Burkholderiales	SC-I-84	SC-I-84	Rare
ASV_30423	27	Connector	Acidobacteriota	Acidobacteriae	Acidobacteriales	uncultured	uncultured	Rare
ASV_30348	11	Connector	Proteobacteria	Alphaproteobacteria	Rhizobiales	Hyphomicrobiaceae	Hyphomicrobium	Rare
ASV_2976	27	Connector	Gemmatimonadota	Gemmatimonadetes	Gemmatimonadales	Gemmatimonadaceae	Gemmatimonas	Rare
ASV_29635	27	Connector	Actinobacteriota	Actinobacteria	Micrococcales	Intrasporangiaceae	unclassified_Intrasporangiaceae	Rare
ASV_28758	N	Connector	Acidobacteriota	Acidobacteriae	Solibacterales	Solibacteraceae	Candidatus_Solibacter	Rare
ASV_2860	31	Connector	Proteobacteria	Gammaproteobacteria	Burkholderiales	Nitrosomonadaceae	MND1	Rare
ASV_2803	N	Connector	Chloroflexi	Chloroflexia	Thermomicrobiales	Thermomicrobiaceae	Sphaerobacter	Rare
ASV_27757	N	Connector	Actinobacteriota	Thermoleophilia	Gaiellales	uncultured	uncultured	Rare
ASV_26490	N	Connector	Actinobacteriota	Actinobacteria	Frankiales	unclassified_Frankiales	unclassified_Frankiales	Rare
ASV_26164	N	Connector	Acidobacteriota	Vicinamibacteria	Vicinamibacterales	Vicinamibacteraceae	Vicinamibacteraceae	Rare

ASV_24879	27	Connector	Acidobacteriota	Vicinamibacteria	Vicinamibacterales	uncultured	uncultured	Rare
ASV_2410	27	Connector	Proteobacteria	Gammaproteobacteria	Xanthomonadales	Rhodanobacteraceae	Chujaibacter	Rare
ASV_23557	11	Connector	Actinobacteriota	Actinobacteria	Frankiales	Geodermatophilaceae	Geodermatophilus	Rare
ASV_23230	10	Connector	Armatimonadota	Armatimonadia	Armatimonadales	Armatimonadales	Armatimonadales	Rare
ASV_22701	N	Connector	Proteobacteria	Alphaproteobacteria	Rhizobiales	KF-JG30-B3	KF-JG30-B3	Rare
ASV_22207	27	Connector	Proteobacteria	Gammaproteobacteria	Burkholderiales	SC-I-84	SC-I-84	Rare
ASV_218	4	Connector	Proteobacteria	Gammaproteobacteria	Burkholderiales	SC-I-84	SC-I-84	Rare
ASV_20174	4	Connector	Gemmatimonadota	Gemmatimonadetes	Gemmatimonadales	Gemmatimonadaceae	Gemmatimonas	Rare
ASV_19941	27	Connector	Chloroflexi	Anaerolineae	SBR1031	A4b	A4b	Rare
ASV_19910	11	Connector	Bacteroidota	Bacteroidia	Flavobacteriales	NS9_marine_group	NS9_marine_group	Rare
ASV_19281	N	Connector	Proteobacteria	Gammaproteobacteria	Burkholderiales	Oxalobacteraceae	unclassified_Oxalobacteraceae	Rare
ASV_18941	11	Connector	Bacteroidota	Bacteroidia	Chitinophagales	Chitinophagaceae	uncultured	Rare
ASV_17524	N	Connector	Actinobacteriota	Thermoleophilia	Gaiellales	uncultured	uncultured	Rare
ASV_16771	10	Connector	Firmicutes	Clostridia	Clostridiales	Clostridiaceae	Clostridium_sensu_stricto_13	Rare
ASV_16314	10	Connector	Actinobacteriota	Actinobacteria	Frankiales	Geodermatophilaceae	Geodermatophilus	Rare
ASV_16139	N	Connector	Actinobacteriota	Actinobacteria	Streptomycetales	Streptomycetaceae	Streptomyces	Rare
ASV_15579	N	Connector	Myxococcota	Polyangia	Polyangiales	Sandaracinaceae	uncultured	Rare
ASV_14917	N	Connector	Acidobacteriota	Vicinamibacteria	Vicinamibacterales	Vicinamibacteraceae	Vicinamibacteraceae	Rare
ASV_14806	N	Connector	Patescibacteria	Saccharimonadia	Saccharimonadales	Saccharimonadales	Saccharimonadales	Rare
ASV_13507	31	Connector	Actinobacteriota	Thermoleophilia	Solirubrobacterales	67-14	67-14	Rare
ASV_13049	10	Connector	Bacteroidota	Bacteroidia	Sphingobacteriales	AKYH767	AKYH767	Rare
ASV_1256	2	Connector	Chloroflexi	Ktedonobacteria	C0119	C0119	C0119	Rare
ASV_11834	11	Connector	Gemmatimonadota	Gemmatimonadetes	Gemmatimonadales	Gemmatimonadaceae	uncultured	Rare
ASV_11566	N	Connector	Actinobacteriota	Actinobacteria	Propionibacteriales	Nocardioidaceae	Nocardioides	Rare
ASV_110106	N	Connector	Gemmatimonadota	S0134_terrestrial_group	S0134_terrestrial_group	S0134_terrestrial_group	S0134_terrestrial_group	Rare
ASV_10981	N	Connector	Firmicutes	Limnochordia	Limnochordia	Limnochordia	Hydrogenispora	Rare
ASV_109800	17	Connector	Proteobacteria	Gammaproteobacteria	Burkholderiales	SC-I-84	SC-I-84	Rare
ASV_109756	27	Connector	Acidobacteriota	Vicinamibacteria	Vicinamibacterales	uncultured	uncultured	Rare

ASV_109587	27	Connector	Chloroflexi	Ktedonobacteria	C0119	C0119	C0119	Rare
ASV_109581	N	Connector	Planctomycetota	Phycisphaerae	Phycisphaerales	Phycisphaeraceae	I-8	Rare
ASV_109450	N	Connector	Acidobacteriota	Acidobacteriae	Solibacterales	Solibacteraceae	Candidatus_Solibacter	Rare
ASV_109403	17	Connector	Dependentiae	Babeliae	Babeliales	Vermiphilaceae	Vermiphilaceae	Rare
ASV_109207	N	Connector	Acidobacteriota	Vicinamibacteria	Vicinamibacterales	uncultured	uncultured	Rare
ASV_10900	N	Connector	Firmicutes	Bacilli	Bacillales	Bacillaceae	uncultured	Rare
ASV_107278	10	Connector	Proteobacteria	Gammaproteobacteria	Xanthomonadales	Xanthomonadaceae	Thermomonas	Rare
ASV_107272	N	Connector	Patescibacteria	Saccharimonadia	Saccharimonadales	LWQ8	LWQ8	Rare
ASV_106588	17	Connector	Gemmatimonadota	Gemmatimonadetes	Gemmatimonadales	Gemmatimonadaceae	Gemmatimonas	Rare
ASV_106149	17	Connector	Proteobacteria	Gammaproteobacteria	Burkholderiales	Oxalobacteraceae	Massilia	Rare
ASV_105625	11	Connector	Bacteroidota	Bacteroidia	Chitinophagales	Chitinophagaceae	uncultured	Rare
ASV_105466	N	Connector	Acidobacteriota	Vicinamibacteria	Vicinamibacterales	uncultured	uncultured	Rare
ASV_105164	11	Connector	Gemmatimonadota	Gemmatimonadetes	Gemmatimonadales	Gemmatimonadaceae	Gemmatimonas	Rare
ASV_10486	11	Connector	Proteobacteria	Gammaproteobacteria	Xanthomonadales	Xanthomonadaceae	Lysobacter	Rare
ASV_104538	N	Connector	Planctomycetota	Phycisphaerae	Phycisphaerales	Phycisphaeraceae	SM1A02	Rare
ASV_104010	31	Connector	Chloroflexi	Chloroflexia	Thermomicrobiales	JG30-KF-CM45	JG30-KF-CM45	Rare
ASV_104005	N	Connector	Verrucomicrobiota	Verrucomicrobiae	Verrucomicrobiales	Verrucomicrobiaceae	uncultured	Rare
ASV_103767	N	Connector	Actinobacteriota	Actinobacteria	Frankiales	Nakamurellaceae	Nakamurella	Rare
ASV_102996	N	Connector	Chloroflexi	Ktedonobacteria	C0119	C0119	C0119	Rare
ASV_102663	11	Connector	Firmicutes	Bacilli	Bacillales	Bacillaceae	Bacillus	Rare
ASV_102164	10	Connector	Actinobacteriota	Thermoleophilia	Gaiellales	uncultured	uncultured	Rare
ASV_101559	N	Connector	Gemmatimonadota	Gemmatimonadetes	Gemmatimonadales	Gemmatimonadaceae	Gemmatimonas	Rare

Table S8. Number, proportion, and mean abundance of abundant and rare ASVs in the two treatment groups, as determined by Zi-Pi screening.

	Outside			In		
	ASV numbers	Poportion of ASVs (%)	Mean relative abundance (%)	ASV numbers	Poportion of ASVs (%)	Mean relative abundance (%)
Abundant	59	28.1	4.58	136	37.7	7.37
Rare	151	71.9	1.81	225	62.3	0.87
Total	210	100	6.39	361	100	8.24

Table S9. Top 20 key ASVs and their associated information identified by random forest analysis in the In treatment.

Label	Module	Zi-Pi-Type	Type	Phylum	Family	Genus	T0	T4	T8	T12
							Relative abundance (%)			
ASV_9659	31	Connectors	Abundant	Actinobacteriota	Micrococcaceae	*unclassified_ Micrococcaceae*	1.417	0.650	0.196	0.826
ASV_91531	31	Connectors	Rare	Gemmatimonadota	Gemmatimonadaceae	*uncultured*	0	0.003	0.004	0
ASV_86559	N	Connectors	Rare	Chloroflexi	JG30-KF-AS9	*JG30-KF-AS9*	0	0	0.006	0.032
ASV_83083	11	Connectors	Abundant	Gemmatimonadota	Gemmatimonadaceae	*Gemmatimonas*	0.018	0.023	0	0.007
ASV_82598	11	Connectors	Abundant	Bacteroidota	Chitinophagaceae	*Flavisolibacter*	0.013	0.010	0.116	0.013
ASV_78498	31	Module hubs	Abundant	Actinobacteriota	67-14	*67-14*	0.037	0	0	0.091
ASV_6828	N	Connectors	Rare	Proteobacteria	Nitrosomonadaceae	*Ellin6067*	0.011	0.013	0.001	0.009
ASV_67865	11	Connectors	Abundant	Chloroflexi	A4b	*A4b*	0.021	0	0.077	0.020
ASV_5906	10	Connectors	Rare	Proteobacteria	TRA3-20	*TRA3-20*	0.017	0	0.035	0.007
ASV_57264	N	Connectors	Abundant	Proteobacteria	A21b	*A21b*	0.021	0.007	0.076	0
ASV_51196	10	Connectors	Rare	Firmicutes	Planococcaceae	*Sporosarcina*	0.032	0.023	0.021	0
ASV_44239	N	Connectors	Rare	Proteobacteria	Moraxellaceae	*Acinetobacter*	0.007	0	0.005	0.015
ASV_44014	N	Connectors	Abundant	Actinobacteriota	Nocardioidaceae	*Nocardioides*	0.004	0.004	0	0.069
ASV_40503	31	Connectors	Abundant	Chloroflexi	B10-SB3A	*B10-SB3A*	0.084	0.076	0.027	0.107
ASV_309	31	Connectors	Rare	Bdellovibrionota	Bdellovibrionaceae	*Bdellovibrio*	0	0.006	0.021	0
ASV_30639	11	Module hubs	Rare	Actinobacteriota	Micrococcaceae	*unclassified_ Micrococcaceae*	0	0	0.013	0.044
ASV_2860	31	Connectors	Rare	Proteobacteria	Nitrosomonadaceae	*MND1*	0.004	0.006	0.012	0
ASV_23557	11	Connectors	Rare	Actinobacteriota	Geodermatophilaceae	*Geodermatophilus*	0.012	0.014	0.005	0
ASV_21711	11	Module hubs	Abundant	Actinobacteriota	Intrasporangiaceae	*unclassified_ Intrasporangiaceae*	0.072	0.196	0	0
ASV_106588	17	Connectors	Rare	Gemmatimonadota	Gemmatimonadaceae	*Gemmatimonas*	0.008	0.003	0.003	0

Table S10. Top 20 key ASVs and their associated information identified by random forest analysis in the Outside treatment.

Label	Module	Zi-Pi-Type	Type	Phylum	Family	Genus	T0	T4	T8	T12
							Relative abundance(%)			
ASV_9950	14	Connectors	Abundant	Proteobacteria	Devosiaceae	*Devosia*	0.505	0.432	0.525	0.489
ASV_96659	21	Connectors	Rare	Actinobacteriota	Nocardioidaceae	*Marmoricola*	0	0.002	0	0.001
ASV_8938	N	Connectors	Abundant	Chloroflexi	Thermomicrobiaceae	*Sphaerobacter*	0	0.011	0	0.019
ASV_88965	14	Connectors	Rare	Firmicutes	Bacillaceae	*Bacillus*	0.009	0.001	0	0.003
ASV_845	N	Connectors	Rare	Proteobacteria	Xanthomonadaceae	*Lysobacter*	0	0	0.002	0.007
ASV_57690	12	Connectors	Rare	Bacteroidota	Microscillaceae	*uncultured*	0.008	0.042	0	0.052
ASV_57024	21	Connectors	Abundant	Chloroflexi	AKYG1722	*AKYG1722*	0.027	0	0.033	0
ASV_55622	N	Connectors	Rare	Acidobacteriota	Vicinamibacteraceae	*Vicinamibacteraceae*	0.002	0	0.006	0
ASV_54409	12	Connectors	Rare	Actinobacteriota	Micromonosporaceae	*unclassified_ Micromonosporaceae*	0.015	0	0.038	0
ASV_53853	14	Connectors	Rare	Acidobacteriota	Subgroup_7	*Subgroup_7*	0.012	0	0.015	0.046
ASV_50257	N	Connectors	Rare	Actinobacteriota	Nocardioidaceae	*Nocardioides*	0.009	0.003	0.006	0.004
ASV_50216	15	Connectors	Rare	Actinobacteriota	67-14	*67-14*	0.027	0.007	0.034	0.065
ASV_50068	14	Connectors	Rare	Proteobacteria	Xanthobacteraceae	*unclassified_ Xanthobacteraceae*	0	0.003	0.002	0
ASV_47667	21	Connectors	Rare	Proteobacteria	Oxalobacteraceae	*Noviherbaspirillum*	0	0.008	0	0.003
ASV_4145	N	Connectors	Rare	Acidobacteriota	Solibacteraceae	*Candidatus_Solibacter*	0	0.012	0.006	0.019
ASV_36013	15	Connectors	Rare	Proteobacteria	Rhizobiaceae	*unclassified_ Rhizobiaceae*	0	0	0.015	0.040
ASV_35636	12	Connectors	Rare	Myxococcota	BIrii41	*BIrii41*	0.054	0	0.052	0
ASV_30438	12	Connectors	Rare	Actinobacteriota	Solirubrobacteraceae	*Conexibacter*	0.046	0.020	0.020	0
ASV_29973	14	Connectors	Abundant	Actinobacteriota	unclassified_Actinobacteria	*unclassified_ Actinobacteria*	0.062	0	0	0.035
ASV_12533	15	Connectors	Rare	Cyanobacteria	Sericytochromatia	*Sericytochromatia*	0.037	0	0.017	0

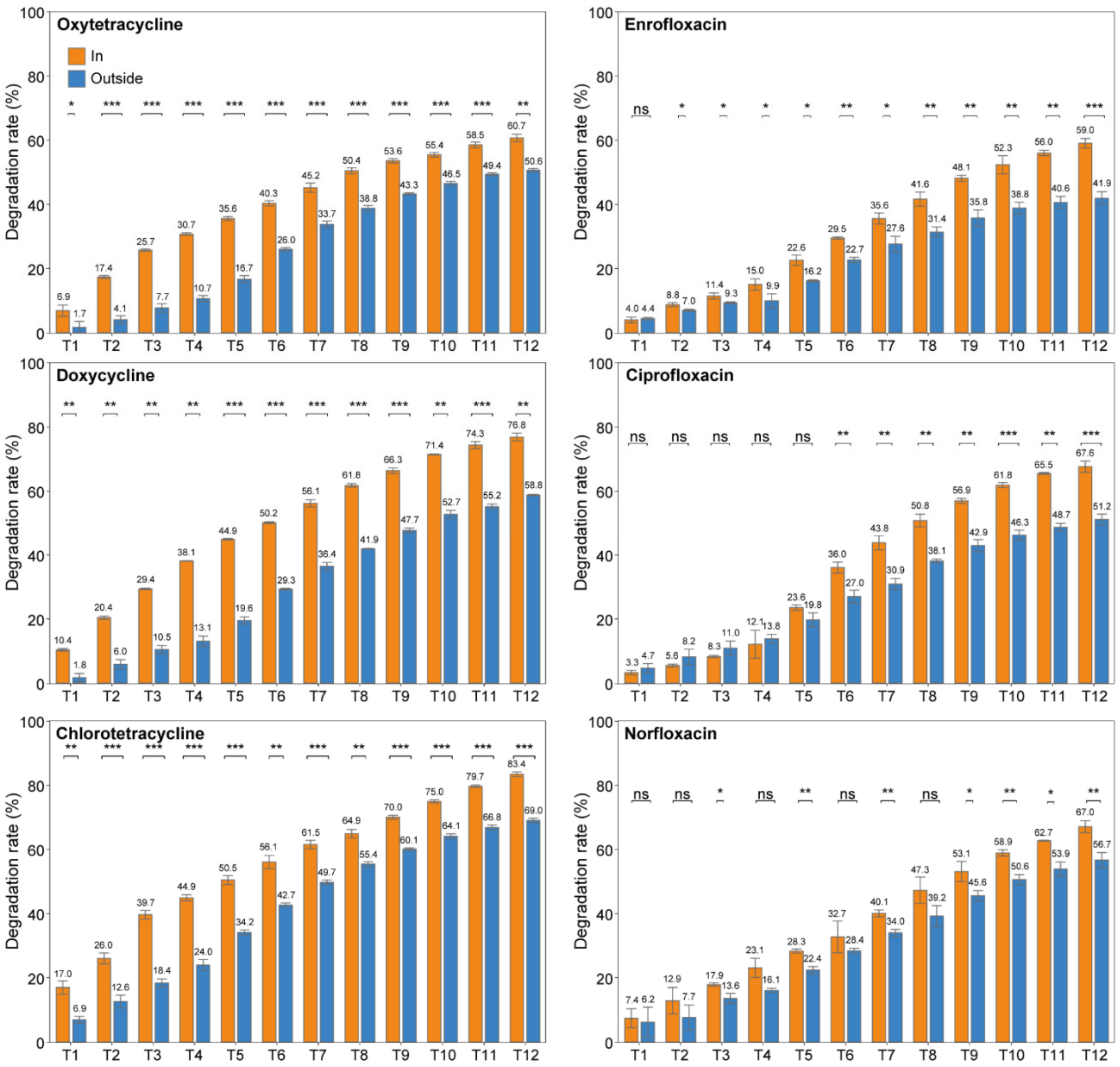

Figure S1. Degradation rate of antibiotics under two treatments during the experiment.

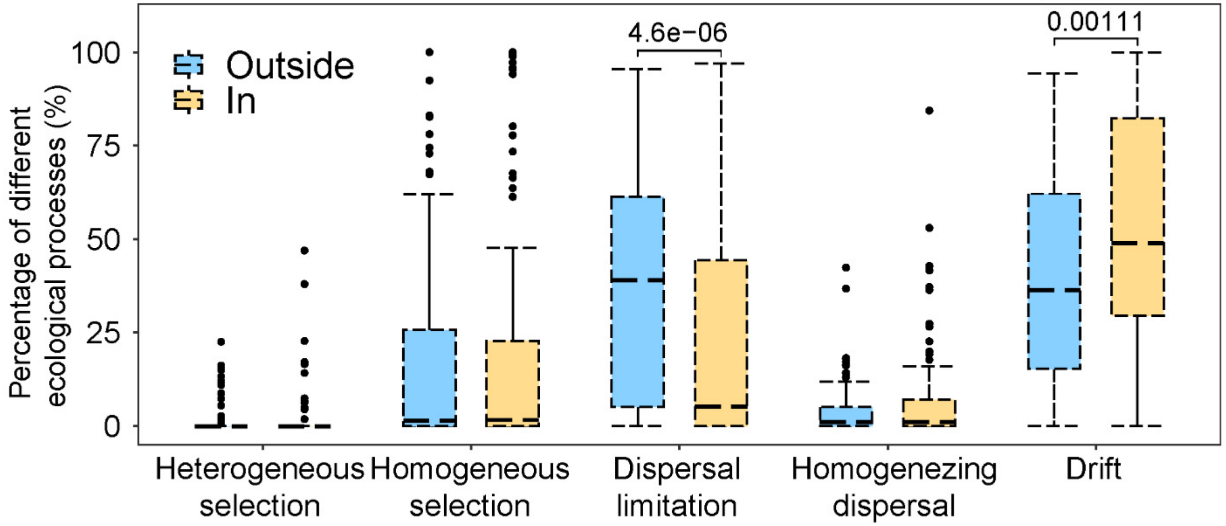

Figure S2. Differences in the relative importance of different ecological processes for the 121 bins between the two treatments. Comparisons were made using the Wilcoxon rank-sum test.
